# Factors Associated With Childhood Undernutrition in Sub‐Saharan Africa: A Systematic Review and Meta‐Analysis

**DOI:** 10.1111/mcn.70083

**Published:** 2025-08-31

**Authors:** Hannah Ricci, Daniela Schmid, Salome Kruger, Stefano Terzoni, Cristian Ricci

**Affiliations:** ^1^ Africa Unit for Transdisciplinary Health Research (AUTHeR) North‐West University Potchefstroom South Africa; ^2^ Faculty of Life Science Albstadt‐Sigmaringen University Sigmaringen Germany; ^3^ Centre of Excellence for Nutrition North‐West University Potchefstroom South Africa; ^4^ Dipartimento Scienze Biomediche per la Salute Università degli Studi di Milano Milan Italy

**Keywords:** infants and young children, stunting, Sub‐Saharan Africa, undernutrition, underweight, wasting

## Abstract

Undernutrition increases the risk of morbidity and mortality, making it essential to identify and address its key determinants. This systematic review and meta‐analysis examines the impact of selected child‐related, household and hygiene, and sanitation factors on the nutritional status of infants and young children aged 0–59 months in Sub‐Saharan Africa. We conducted a comprehensive search of online databases using defined Medical Subject Headings and keyword search terms. Nutritional status was assessed using the WHO child growth Standards *Z*‐scores for stunting, underweight and wasting. A meta‐analysis was performed to estimate pooled associations, and heterogeneity was assessed using the Cochrane *Q* and *I* ^2^ statistic. Sensitivity analyses were conducted, and publication bias was evaluated. Out of 1, 992 articles identified, 49 studies met the inclusion criteria. Our findings indicated that diarrhoea was associated with 77% increased risk of overall undernutrition (odds ratio (OR) = 1.77; confidence interval [Cl] = 1.52, 2.06), and 92% increased risk of wasting (OR = 1.92; 95% Cl = 1.48, 2.48). Low birthweight was linked to a two‐fold increased risk of stunting (OR = 2.35; 95% Cl = 1.84, 3.00), while low maternal education was associated with a higher risk of underweight (OR = 1.55; 95% Cl = 1.17, 2.04). These findings highlight the need for targeted interventions to reduce childhood undernutrition in the region.

## Summary

- Undernutrition remains a significant public health issue among infants and young children aged 0–59 months in Sub‐Saharan Africa.
- We identified factors that are consistently associated with undernutrition in infants and young children aged 0–59 months in Sub‐Saharan Africa.
- Diarrhoea, low birthweight and low maternal education were concurrently associated with overall undernutrition, stunting, underweight and wasting in infants and young children aged 0–59 months in Sub‐Saharan Africa.

## Graphical abstract

Key child, household and hygiene factors—especially diarrhoea, low birthweight and low maternal education—significantly increase the risk of undernutrition, including stunting, underweight and wasting in young children across Sub‐Saharan Africa, underscoring the need for targeted interventions.

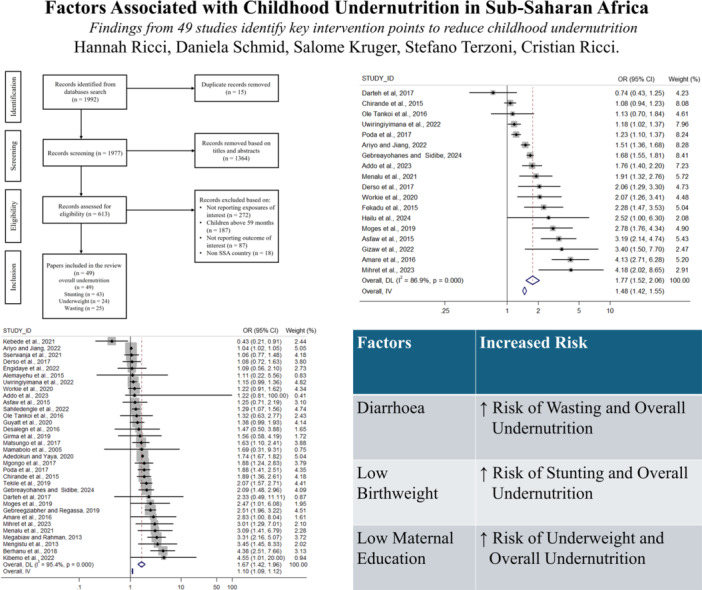

## 1 Introduction

Undernutrition, defined as inadequate intake of energy and nutrients (Maleta 2006), remains a significant public health issue among infants and young children aged 0–59 months, particularly in Sub‐Saharan Africa, where it contributes to 45% of deaths in this age group (Black et al. 2013; WHO 2024b). It manifests in four key forms: Stunting (low height‐for‐age), underweight (low weight‐for‐age), wasting (low weight‐for‐height) and micronutrient deficiencies (insufficient essential vitamins and minerals for optimal health) (Maleta 2006). Globally, 22.3% of infants and young children aged 0–59 months are stunted, 6.8% are wasted and 15.7% are underweight (UNICEF, WHO, & WB Group 2012; UNICEF, W. W. G 2023). Progress in reducing childhood undernutrition, particularly stunting, has been slow, with an annual reduction of 1.65% since 2012—well below the 6.08% needed to meet the 2030 Sustainable Development Goals (UNICEF, W. W. G 2023). If current trends persist, an estimated 19.5% of infants and young children aged 0–59 months will be stunted by 2030, with Africa bearing a significant burden as home to two‐fifths of the world's stunted children and over a quarter of those who are wasted (UNICEF, W. W. G 2023). This persistent burden highlights the urgent need to understand not just the prevalence but also the complexity of factors driving undernutrition within this region.

Childhood undernutrition in low‐ and middle‐income countries is influenced by multiple interrelated factors categorised as immediate, underlying and basic determinants (Reinhardt and Fanzo 2014). Immediate factors include inadequate dietary intake, such as poor breastfeeding practices and low micronutrient content in complementary foods, as well as illnesses like diarrhoea, which further deplete essential nutrients and impair growth (Reinhardt and Fanzo 2014). Underlying factors encompass household food insecurity, inadequate feeding practices and poor access to safe water, hygiene and sanitation—all of which are critical for child health and development (Reinhardt and Fanzo 2014; Stewart et al. 2013; UNICEF, WHO, & WB Group 2021). At the foundational level, determinants such as limited financial resources, low education levels and insufficient human capital further exacerbate undernutrition (UNICEF, WHO, & WB Group 2021). These factors collectively undermine household capacity to meet children's nutritional needs, placing the most vulnerable at heightened risk (UNICEF, WHO, & WB Group 2021). Importantly, these determinants are not isolated; they often co‐occur and interact in complex ways, which can amplify their effects on child nutrition.

The consequences of childhood undernutrition extend beyond immediate health concerns. Undernourished children face higher risks of infections, increased morbidity and mortality, impaired physical growth and delayed cognitive development. These effects, in turn, hinder academic performance and reduce long‐term economic productivity. Given these far‐reaching implications, a comprehensive understanding of undernutrition's determinants is essential for informing effective interventions (Black et al. 2008; Black et al. 2013). In addition, addressing these determinants can contribute to achieving multiple development goals, including those related to education, equity and economic growth.

Despite global efforts, reducing childhood undernutrition has proven challenging, with progress remaining insufficient to meet global targets (UN 2023; UNICEF, W. W. G 2023; WHO 2014). While numerous studies have explored undernutrition in Sub‐Saharan Africa, most have focused on isolated determinants, limiting a holistic understanding of how these factors interact. Individual studies (Adedokun and Yaya 2021; Ararsa et al. 2023; Gebreayohanes and Dessie 2022; Mihret et al. 2023; Rezaeizadeh et al. 2024) and systematic reviews (Adamou et al. 2024; Asebe et al. 2024; Kerac et al. 2019; Tesema et al. 2021; Worku et al. 2025) have examined some of these determinants, but many focus on specific risk factors or a single outcome measure, limiting broader applicability across diverse contexts. In addition, previous studies vary in study design, sample sizes, geographic area and methodological rigour, making it difficult to generalise findings across the entire region. Moreover, few reviews have systematically examined whether the same risk factors are consistently associated with multiple undernutrition outcomes. While some studies have explored overlapping outcomes such as stunting and wasting (Kerac et al. 2019), they typically focus on a limited range of determinants, leaving a gap in the evidence needed for prioritising cross‐cutting interventions.

This systematic review and meta‐analysis addresses these research gaps by synthesising evidence from multiple studies to provide a comprehensive assessment of how 14 child‐related, household and hygiene determinants are individually associated with undernutrition outcomes, including stunting, underweight and wasting. Furthermore, we explore patterns across multiple undernutrition outcomes. Identifying factors that are consistently associated with more than one form of undernutrition, offering insights into common risk factors across different forms of undernutrition to help identify actionable intervention strategies. To our knowledge, this is one of the few reviews to examine how common risk factors are linked to multiple undernutrition outcomes, providing insights that may help guide the development of integrated interventions. This approach provides robust, evidence‐based insights to guide policy and programme development aimed at reducing childhood undernutrition in Sub‐Saharan Africa.

## 2 Methods

This study is based on the Preferred Reporting Items for Systematic Reviews and Meta‐Analyses (PRISMA) guidelines (Page et al. 2021), while the PECO (ST) criteria, where P stands for Population, E for Exposure, C for Comparison, O for Outcome and ST for the Setting and/or Time (Morgan et al. 2018) were used to compute the search string to identify the studies involved. The target population comprised infants and young children aged 0–59 months. The specific exposures included child‐related factors (anaemia, birthweight, breastfeeding, diarrhoea and micronutrient supplementation), household factors (area of residence [urban vs. rural], family size, food security, maternal education, number of children and socioeconomic status) and hygiene and sanitation factors (handwashing, toilet facility and water source). These factors were selected because they are modifiable or partially modifiable risk factors that have been consistently reported in the literature as being associated with childhood undernutrition. In addition, these factors are amenable to intervention and can be targeted through public health strategies, making them particularly relevant for addressing undernutrition in Sub‐Saharan Africa (Ricci et al. 2019; Ricci et al. 2019). In this study, we specifically aimed to synthesise existing evidence and identify key modifiable risk factors that can inform public health interventions. The outcome was overall undernutrition, stunting, underweight and wasting. This is a systematic review and meta‐analysis of observational studies conducted in Sub‐Saharan Africa and published from 1 January 2004 to 30 September 2024. The literature search for the systematic review was done using PubMed, ScienceDirect, Web of Science, Cochrane Library and the Cumulative Index to Nursing and Allied Health Literature. Potentially eligible studies were also identified by screening the reference lists of original studies, and previous systematic reviews and meta‐analyses. This study was registered in the PROSPERO International prospective register of systematic reviews (CRD42024512160).

### 2.1 Search Strategy

Our online database search involved defining Medical Subject Headings (MeSH) terms and using keyword search terms defined according to the PECO (ST) criteria. Each item of the PECO (ST) was associated with a variable number of MeSH terms linked by the OR Boolean operator. Subsequently, each of the strings aimed to identify a given PECO (ST) criterion was linked together using the AND Boolean operator, while the NOT Boolean operator was used to avoid the over‐selection of ineligible studies (Supporting Information S1). Furthermore, we applied language restriction to limit our database search to English‐language peer‐reviewed academic journals.

### 2.2 Selection Process

Studies were included if they were population‐ or community‐based observational studies. In addition, we included studies that reported the odds ratio (OR) or risk ratios with their corresponding 95% confidence intervals (95% Cl), comparing high versus low levels or levels of interest for any of the above‐mentioned factors’ association with undernutrition, with the most fully adjusted exposure factor values in relation to the outcome being extracted. Identified studies used length/height‐for‐age, weight‐for‐age and weight‐for‐length/height *Z*‐scores below −2 standard deviation from the median of the World Health Organisation Child Growth Standards (WHO 2006) to assess child growth outcomes, including stunting, underweight and wasting, as well as directly identifying these conditions as the specific outcomes. Identified records were first screened by deleting all duplicates and unrelated articles, followed by screening all titles and abstracts for potentially eligible studies. The full texts of potentially eligible studies were then screened independently by two reviewers for final eligibility for inclusion or exclusion. Any discrepancies regarding the eligibility of a paper for inclusion or exclusion were resolved by consensus or by consulting a third reviewer. Studies were excluded if they were randomised controlled trials, commentaries, editorials, reviews, published figures, reports, patents, theses, posters, letters, conferences and seminars. The following information was extracted from eligible studies after the screening: (1) the first author's surname, (2) year of publication, (3) geographical location, (4) study design, (5) sample size, (6) participant population, (7) mean or median age of the children, (8) sex, (9) frequency of undernutrition, (10) method of assessment of undernutrition (including stunting, underweight and wasting) and (11) the exposure (factors associated with undernutrition, including stunting, underweight and wasting).

### 2.3 Quality Assessment

The quality of the included studies was assessed by two appraisers using the Joanna Briggs Institute (JBI) critical appraisal tool for analytical cross‐sectional studies. This tool uses a tick system to appraise eight items based on the inclusion and exclusion criteria, subjects and setting, exposure, measurement of the condition, confounders, outcomes measurement and statistical analysis (JBI 2020). A score of one was assigned if the response to a criterion was ‘yes’, and a score of zero was assigned if the response to a criterion was ‘no’ or ‘unclear’. The individual points were computed and reported as total quality scores.

### 2.4 Statistical Methods

In this study, we conducted a random‐effect meta‐analysis using inverse variance weighting to combine OR or relative risk estimates of undernutrition for categories of high versus low risk of undernutrition for each factor under investigation. Specifically, the weight of the *i*th study was calculated as *w*_i_ = 1/(*s_i_* ^2^ + *t* ^2^), where *s_i_* ^2^ represents the variance estimate from that study, and *t* ^2^ is the overall variance. Between‐study heterogeneity was assessed using the *I* ^2^ statistic, which quantifies the percentage of total variation across studies due to heterogeneity, with higher values indicating greater variability in study results (Higgins 2003) and tested for statistical significance using the Cochrane *Q* test, a chi‐squared test for heterogeneity, was also used, with significant results indicating variations across studies that cannot be explained by chance alone. Heterogeneity was considered relevant if the *I* ^2^ statistic exceeded 50% or if the Cochrane *Q* test was statistically significant at the 5% threshold. In addition, to assess the robustness of the results, an influence analysis was conducted by sequentially removing one study at a time to assess its impact on the overall results. Publication bias was assessed by visual inspection of the funnel plot and by performing Egger's test, with visual inspection used exclusively when fewer than five studies were included (Egger et al. 1997). A two‐tailed Type‐I error rate of 10% was used to determine potential publication bias. For the overall undernutrition risk estimates, a fixed‐effects meta‐analysis was applied to merge estimates for different types of undernutrition (stunting, underweight, or wasting). Similarly, the fixed‐effects approach was used to merge within‐study estimates when a study reported multiple outcomes. All analyses were conducted using STATA 14 statistical software, with the METAN, METANINF, METABIAS, METAFUNNEL and METAREG functions used to combine results, perform influence analysis, assess publication bias, generate funnel plots and conduct meta‐regression.

## 3 Results

### 3.1 Study Characteristics

**Figure 1:**
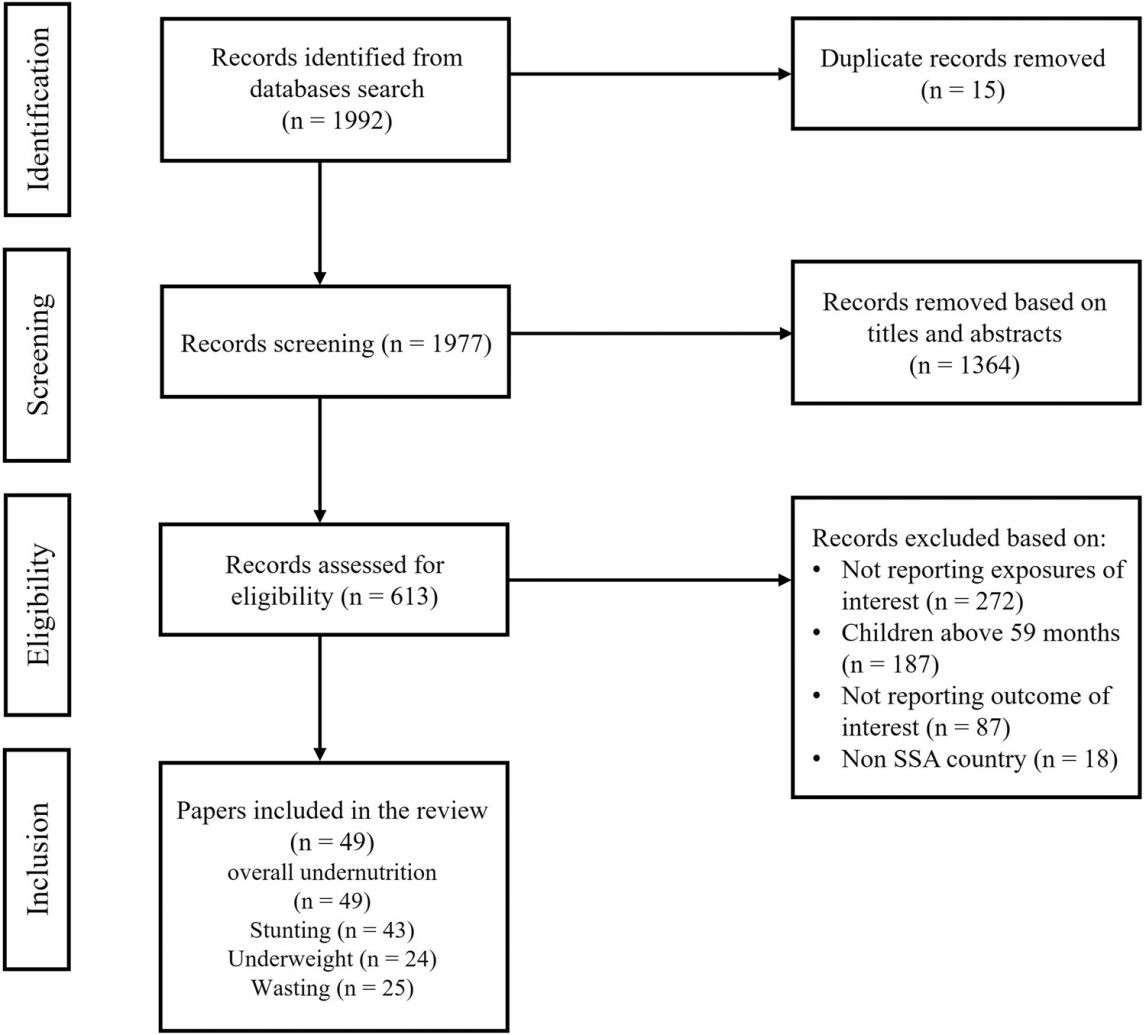
PRISMA diagram for study selection.

Figure 1 presents the PRISMA diagram outlining the study selection process. From our initial search, we identified 1992 records. After removing duplicates, 1981 records were screened for title and abstract. Following a thorough review of 613 full‐text articles, 49 studies met the eligibility criteria for inclusion in this systematic review and meta‐analysis. The study characteristics are summarised in Table 1. The median publication year of the included studies was 2019, spanning a range from 2005 (Mamabolo et al. 2005) to 2024 (Gebreegziabher and Sidibe 2024). Of the 49 studies, 37 were conducted in Eastern Africa, predominantly in Ethiopia, 81.1% (*n* = 30). The remaining studies were distributed across Western Africa (seven studies), Southern Africa (two studies) and Central Africa (two studies), with one study covering all of Sub‐Saharan Africa (Adedokun and Yaya 2021).

**Table 1:** Characteristics of the included studies^a^.

		Sample size		Mean age, months			Baseline prevalence %			Quality scores
Author	Country		Participants		Male %	Female %	Stunting	Underweight	Wasting	
Addo et al. (2023)	Benin	13,589	0–59 months	NR	51	49	31.6	16.7	5.0	4
Adedokun and Yaya (2021)	SSA	189,195	0–59 months	NR	NR	NR	26	21	6	4
Alemayehu et al. (2015)	Ethiopia	605	0–59 months	32.1 ± 17.3	49.4	50.6	56.6	45.3	34.6	6
Amare et al. (2016)	Ethiopia	342	0–59 months	28.9 ± 16.4	50.9	49.1	24.9	14.3	11.1	6
Ararsa et al. (2023)	Ethiopia	580	6–23 months	NR	51	49	32.1	9	7	8
Ariyo and Jiang (2022)	Nigeria	11,471	3–59 months	29.9 ± 16.5	51.3	48.7	38.8	22.4	7.1	5
Asa et al. (2022)	Cameroon	422	12–59 months	NR	43.84	56.2	22.2	32.9	28.6	6
Asfaw et al. (2015)	Ethiopia	778	6 –59 months	28 ± 14.6	49.4	50.6	47.6	29.1	13.4	8
Berhanu et al. (2018)	Ethiopia	1039	24–59 months	40.9 ± 10.4	44.4	55.6	39.3	NR	NR	7
Bliznashka et al. (2021)	Nigeria	285	0–59 months	32.9 ± 5	NR	NR	18.6	29.5	25.3	5
Chirande et al. (2015)	Tanzania	7234	0–59 months	NR	49.8	50.2	41.6	NR	NR	5
Darteh et al. (2017)	Ghana	2720	0–59 months	NR	47.6	52.4	NR	NR	5	5
Derso et al. (2017)	Ethiopia	587	6–24 months	15 ± 5.7	96.9	3.1	58.1	NR	17	7
Desalegn et al. (2016)	Ethiopia	312	6–59 months	NR	50.3	49.7	26.6	NR	NR	5
Dewana et al. (2017)	Ethiopia	764	24–59 months	NR	53	47	52.5	NR	NR	6
Engidaye et al. (2022)	Ethiopia	432	6–59 months	24 (14–42)^d^	41.2	58.8	50.2	28	11.3	6
Fekadu et al. (2015)	Ethiopia	214	6–23 months	15.4 ± 6	51.9	48.1	22.9	19.5	17.5	5
García Cruz et al. (2017)^b^	Mozambique	282	0–59 months	41.8 ± 18.3	57.4	42.6	NR	NR	NR	7
Gebreayohanes and Dessie (2022)	Ethiopia	554	6–59 months	31 (19)^d^	56.3	43.7	39.5	NR	NR	6
Gebreayohanes and Dessie (2022)	Mali	8908	0–59 months	NR	51	49	22.2	9.1	23.6	4
Gebreegziabher and Regassa (2019)	Ethiopia	9696	0–59 months	NR	50.9	49.1	39.2	24.1	10.6	5
Gebru et al. (2019)	Ethiopia	394	0–59 months	NR	43.7	56.3	49.2	NR	NR	4
Girma et al. (2019)	Ethiopia	401	24–59 months	41.2 ± 10.6	50.4	49.6	28.4	13.4	10	7
Gizaw et al. (2022)	Ethiopia	224	24–59 months	43 ± 13	49.1	50.9	33	NR	NR	6
Guyatt et al. (2020)	Kenya	1004	0–23 months	12	50	50	23	10	6	6
Hailu et al. (2024)	Ethiopia	396	6–23 months	14.7 ± 4.4	48.9	51.1	NR	NR	NR	6
Kebede et al. (2021)	Ethiopia	974	0–59 months	NR	51.3	48.7	46.3	28.4	9.8	5
Kibemo et al. (2022)	Ethiopia	340	6–59 months	29.5 ± 24	50.3	49.7	14.7	4.4	2.1	7
Mamabolo et al. (2005)^c^	South Africa	162	36 months	NR	NR	NR	48	9	1	6
Matsungo et al. (2017)	South Africa	750	6 months	6·2 ± 0·3	51.6	48.4	28.5	11.1	1.7	8
Zeleke and Rahman (2013)	Ethiopia	9611	0–59 months	29.4 ± 17.3	50.9	49.1	42.3	NR	NR	3
Menalu et al. (2021)	Ethiopia	385	0–59 months	NR	49.9	50.1	41	26	33	6
Mengesha et al. (2021)	Ethiopia	615	0–59 months	25.6 ± 14.3	46.8	53.2	37.7	NR	NR	6
Mengistu et al. (2013)	Ethiopia	820	6–59 months	32.1 ± 14.9	50	50	47.6	30.9	16.7	7
Mgongo et al. (2017)	Tanzania	1870	0–24 months	12.2 ± 7.8	52	48	41.9	46	24.7	6
Mihret et al. (2023)	Ethiopia	504	6–59 months	25 ± 12	47.2	52.8	NR	NR	13.9	6
Moges et al. (2019)	Ethiopia	415	6–59 months	NR	52.8	47.2	48.4	NR	NR	6
Ole Tankoi et al. (2016)	Kenya	350	6–59 months	NR	54.9	45.1	31	22	8	8
Poda et al. (2017)	Burkina‐Faso	6337	0–59 months	NR	51	49	34.8	25.8	15.7	4
Sahiledengle et al. (2024)	Ethiopia	2146	0–23 months	NR	50.5	49.5	27.2	16.4	7.8	6
Sserwanja et al. (2021)	Sierra Leone	4045	0–59 months	27.1 ± 14.1	50.5	49.5	29.1	NR	NR	5
Beyene Teferi (2016)	Ethiopia	324	6–59 months	27.8 ± 14.8	49.4	50.6	33.3	NR	NR	7
Tekile et al. (2019)	Ethiopia	9495	0–59 months	NR	51.1	48.9	38.3	23.3	10.1	3
Uwiringiyimana et al. (2019)	Rwanda	138	5–30 months	NR	48	52	42	NR	NR	6
Uwiringiyimana et al. (2022)	Rwanda	3593	0–59 months	NR	50.8	49.2	NR	NR	NR	1
Vonaesch et al. (2017)	CAR	422	0–59 months	14.2 ± 10	58	42	36	NR	NR	5
Workie et al. (2020)	Ethiopia	595	12–59 months	33.9 ± 13.9	50.7	49.3	34.1	11.9	6.9	7
Yigezu et al. (2024)	Ethiopia	386	6–59 months	27.11 ± 14.7	51.7	48.3	49	19.7	10.1	7
Yisak et al. (2015)	Ethiopia	791	0–59 months	22.4 ± 1.3	56.8	43.2	45.8	21	10.7	6

Abbreviations: CAR: Central African Republic; NR: not reported; SSA, Sub‐Saharan Africa.

^a^ All studies were cross‐sectional unless otherwise indicated.

^b^ Case‐control study.

^c^ Prospective cohort study.

^d^ Median values reported.

Regarding study design, the majority were cross‐sectional (*n* = 47), with one case‐control study (García Cruz et al. 2017) and one prospective cohort study (Mamabolo et al. 2005). The sample sizes varied from 138 (Uwiringiyimana et al. 2019) to 189,195 (Adedokun and Yaya 2021), with a median sample size of 595. The mean age of the children across studies was approximately 28 ± 15 months, with at least 51% of participants being female. The outcomes related to undernutrition were reported in the following ways: 43 studies reported on stunting, 24 on underweight and 25 on wasting. Two studies (Addo et al. 2023; Hailu et al. 2024) addressed overall undernutrition without separating it by stunting, underweight, or wasting.

### 3.2 Factors Associated With Undernutrition

Table 2 summarises the factors associated with undernutrition. We found significant associations between several factors and the increased risk of undernutrition in children. Diarrhoea, anaemia, low birthweight, low maternal education, large family size and lack of handwashing were all linked to increased risk. Specifically, diarrhoea and anaemia were associated with a 77% (OR = 1.77; 95% CI = 1.52, 2.06) and 72% (OR = 1.72; 95% CI = 1.36, 2.18) increased risk, respectively. Low birthweight (OR = 2.40; 95% CI = 2.03, 2.83) was associated with a two‐fold increase in the risk of undernutrition. Large family size and low maternal education increased the risk by 87% (OR = 1.87; 95% CI = 1.37, 2.56) and 67% (OR = 1.67; 95% CI = 1.42, 1.96), respectively. A higher number of children in the household was linked to a two‐fold increase (OR = 1.46; 95% CI = 1.20, 1.76). Poor hygiene, especially the absence of handwashing, was associated with a 74% (OR = 1.74; 95% CI = 1.13, 2.68) increased risk of undernutrition.

**Table 2:** Factors associated with undernutrition with publication bias.

Exposure	No of studies used	OR (95% Cl)	*I* ^2^ (%)	a *p* value	Publication bias
Child factors					
Anaemia (anaemic vs. non‐anaemic)	4	1.72 (1.36, 2.18)	84.6	< 0.0001	Some form of bias^b^
Birthweight (low vs. high)	12	2.40 (2.03, 2.83)	87.5	< 0.0001	0.001
Breastfeeding (no vs. yes)	19	1.38 (0.89, 2.15)	98.0	< 0.0001	0.213
Diarrhoea (yes vs. no)	18	1.77 (1.52, 2.06)	86.9	< 0.0001	0.080
Supplementation (no vs. yes)	4	1.06 (1.00, 1.13)	0.0	0.431	No bias^b^
Household factors					
Area of residence (rural vs. urban)	15	1.22 (1.07, 1.39)	93.9	< 0.0001	0.763
Family size (high vs. low)	16	1.87 (1.37, 2.56)	85.1	< 0.0001	0.055
Food security (food insecure vs. food secure)	7	1.40 (1.08, 1.82)	69.9	0.0003	0.949
Maternal education (low vs. high)	33	1.67 (1.42, 1.96)	95.4	< 0.0001	0.006
Number of children (high vs. low)	15	1.46 (1.20, 1.76)	87.2	< 0.0001	0.040
Socioeconomic status (poor vs. rich)	29	1.43 (1.23, 1.67)	93.6	< 0.0001	0.122
Hygiene and sanitation					
Handwashing (no vs. yes)	5	1.74 (1.13, 2.68)	53.6	0.071	No bias^b^
Toilet facility (unimproved vs. improved)	19	1.16 (1.05, 1.26)	64.5	< 0.0001	0.570
Water source (unimproved vs. improved)	25	1.21 (1.07, 1.37)	93.7	< 0.0001	0.220

a *Q* test for heterogeneity.

^b^ Funnel plot reported.

### 3.3 Factors Associated With Stunting

Factors associated with stunting are presented in Table 3. Anaemia, diarrhoea, low birthweight, low maternal education, high family size, large number of children in the household, poor socioeconomic status and rural residence were all found to significantly increase the risk of stunting. Anaemia was linked to a 67% (OR = 1.67; 95% CI = 1.27, 2.21) increased risk, and low birthweight showed a two‐fold increased risk (OR = 2.35; 95% CI = 1.84, 3.00). Diarrhoea also increased the risk by 52% (OR = 1.52; 95% CI = 1.31, 1.76). Low maternal education and high number of children were associated with an increased risk of stunting by 68% (OR = 1.68; 95% CI = 1.44, 1.97) and 56% (OR = 1.56; 95% CI = 1.21, 2.02), respectively. Poor socioeconomic status (OR = 1.46; 95% Cl = 1.22, 1.76), and rural residence (OR = 1.35; 95% Cl = 1.11, 1.64) were associated with stunting. Although not statistically significant, high family size was also associated with a 56% (OR = 1.56; 95% CI = 0.98, 2.49) increased risk of stunting.

**Table 3:** Factors associated with stunting with publication bias.

Exposure	No of studies used	OR (95% Cl)	*I* ^2^ (%)	a *p* value	Publication bias
Child factors					
Anaemia (anaemic vs. non‐anaemic)	4	1.67 (1.27, 2.21)	78.9	0.003	No bias^b^
Birthweight (low vs. high)	10	2.35 (1.84, 3.00)	89.6	< 0.0001	0.002
Breastfeeding (no vs. yes)	17	1.39 (0.81, 2.40)	98.0	< 0.0001	0.097
Diarrhoea (yes vs. no)	15	1.52 (1.31, 1.76)	69.7	< 0.0001	0.014
Supplementation (no vs. yes)	5	1.27 (0.83, 1.94)	94.0	< 0.0001	No bias^b^
Household factors					
Area of residence (rural vs. urban)	14	1.35 (1.11, 1.64)	93.1	< 0.0001	0.963
Family size (high vs. low)	11	1.56 (0.98, 2.49)	88.8	< 0.0001	0.808
Food security (food insecure vs. food secure)	6	1.26 (0.91, 1.76)	62.3	0.021	0.515
Maternal education (low vs. high)	30	1.68 (1.44, 1.97)	90.3	< 0.0001	0.001
Number of children (high vs. low)	12	1.56 (1.21, 2.02)	88.3	< 0.0001	0.052
Socioeconomic status (poor vs. rich)	25	1.46 (1.22, 1.76)	91.2	< 0.0001	0.200
Hygiene and sanitation					
Handwashing (no vs. yes)	4	1.52 (0.86, 2.69)	54.1	0.089	No bias^b^
Toilet facility (unimproved vs. improved)	13	1.16 (1.05, 1.28)	40.9	0.062	0.088
Water source (unimproved vs. improved)	20	1.28 (1.03, 1.52)	92.9	< 0.0001	0.160

a *Q* test for heterogeneity.

^b^ Funnel plot reported.

### 3.4 Factors Associated With Underweight

Factors contributing to underweight are shown in Table 4. Diarrhoea, low birthweight, lack of vitamin/mineral supplementation, low maternal education, large family size, food insecurity, lack of handwashing and the use of unimproved water sources all significantly increased the risk of underweight. Diarrhoea was linked to a 92% (OR = 1.92; 95% CI = 1.51, 2.43) increased risk, while low birthweight was associated with more than two‐fold increase in risk (OR = 2.39; 95% CI = 2.00, 2.85). Not receiving vitamin and/or mineral supplementation was also associated with a 16% increased risk (OR = 1.16; 95% CI = 1.06, 1.27). Both large family size (OR = 1.68; 95% CI = 1.07, 2.64) and low maternal education (OR = 1.55; 95% CI = 1.17, 2.04) significantly increased the risk of underweight. In addition, a higher number of children in the household was linked to a 20% increased risk (OR = 1.20; 95% CI = 1.00, 1.45). Household food insecurity was associated with a 68% increased risk of underweight (OR = 1.68; 95% CI = 0.83, 3.38), although this finding was not statistically significant. The absence of handwashing was associated with a three‐fold increased risk (OR = 2.50; 95% CI = 1.31, 4.75), based on a single study (Girma et al. 2019). Finally, low socioeconomic status (OR = 1.33; 95% CI = 1.06, 1.67) and the use of unimproved water sources (OR = 1.17; 95% CI = 1.03, 1.33) were both significantly associated with increased odds of underweight.

**Table 4:** Factors associated with underweight with publication bias.

Exposure	No of studies used	OR (95% Cl)	*I* ^2^ (%)	a *p* value	Publication bias
Child factors					
Anaemia (anaemic vs. non‐anaemic)	3	1.29 (0.91, 1.82)	74.3	0.021	No bias^b^
Birthweight (low vs. high)	8	2.39 (2.00, 2.85)	64	0.007	0.037
Breastfeeding (no vs. yes)	7	1.22 (0.91, 1.63)	62.0	< 0.015	0.943
Diarrhoea (yes vs. no)	8	1.92 (1.51, 2.43)	72.6	0.001	0.035
Supplementation (no vs. yes)	4	1.16 (1.06, 1.27)	0.0	0.783	No bias^b^
Household factors					
Area of residence (rural vs. urban)	10	1.09 (0.95, 1.26)	67.6	0.001	0.458
Family size (high vs. low)	5	1.68 (1.07, 2.64)	66.2	0.019	No bias^b^
Food security (food insecure vs. food secure)	3	1.68 (0.83, 3.38)	46.0	0.157	No bias^b^
Maternal education (low vs. high)	16	1.55 (1.17, 2.04)	95.9	< 0.0001	0.143
Number of children (high vs. low)	8	1.20 (1.00, 1.45)	64.7	0.006	0.117
Socioeconomic status (poor vs. rich)	15	1.33 (1.06, 1.67)	91.9	< 0.0001	0.318
Hygiene and sanitation					
Handwashing (no vs. yes)	1	2.50 (1.31, 4.75)	0.0	—	Some form of bias^b^
Toilet facility (unimproved vs. improved)	9	1.14 (0.99, 1.33)	62.1	0.007	0.663
Water source (unimproved vs. improved)	12	1.17 (1.03, 1.33)	80.4	< 0.0001	0.074

a *Q* test for heterogeneity.

^b^ Funnel plot reported.

### 3.5 Factors Associated With Wasting

Table 5 details the factors associated with wasting. Diarrhoea, anaemia, low birthweight, low maternal education, poor socioeconomic status, rural residence, using unimproved water sources, and not practising handwashing were significant risk factors. Diarrhoea was associated with a 92% increased risk of wasting (OR = 1.92; 95% CI = 1.48, 2.48), while low birthweight was linked to an 86% increased risk (OR = 1.86; 95% CI = 1.60, 2.15). Anaemia was associated with a three‐fold increased risk (OR = 2.50; 95% CI = 1.37, 4.56). Low maternal education (OR = 1.60; 95% CI = 1.19, 2.14) and poor socioeconomic status (OR = 1.35; 95% CI = 1.09, 1.67) were both associated with wasting. Rural residence was also linked to an increased risk (OR = 1.15; 95% CI = 1.02, 1.29). One study (Girma et al. 2019) found that lack of handwashing was associated with an 11‐fold increased risk of wasting (OR = 11.00; 95% CI = 4.34, 27.89).

**Table 5:** Factors associated with wasting with publication bias.

Exposure	No of studies used	OR (95% Cl)	*I* ^2^ (%)	a *p* value	Publication bias
Child factors					
Anaemia (anaemic vs. non‐anaemic)	3	2.50 (1.37, 4.56)	90.3	< 0.0001	No bias^b^
Birthweight (low vs. high)	5	1.86 (1.60, 2.15)	25.9	0.249	No bias^b^
Breastfeeding (no vs. yes)	6	0.98 (0.69, 1.41)	75.7	0.001	0.475
Diarrhoea (yes vs. no)	11	1.92 (1.48, 2.48)	79.2	< 0.0001	0.420
Supplementation (no vs. yes)	4	1.10 (0.77, 1.58)	82.8	0.001	No bias^b^
Household factors					
Area of residence (rural vs. urban)	9	1.15 (1.02, 1.29)	47.1	< 0.067	0.379
Family size (high vs. low)	7	1.10 (0.76, 1.11)	52.4	0.050	0.668
Food security (food insecure vs. food secure)	2	1.33 (0.68, 2.62)	0.0	0.932	Some form of bias^b^
Maternal education (low vs. high)	14	1.60 (1.19, 2.14)	91.2	< 0.0001	0.105
Number of children (high vs. low)	8	1.04 (0.96, 1.13)	0.0	0.723	0.453
Socioeconomic status (poor vs. rich)	15	1.35 (1.09, 1.67)	83.8	< 0.0001	0.119
Hygiene and sanitation					
Handwashing (no vs. yes)	1	11.00 (4.34, 27.89)	0.0	—	Some form of bias^b^
Toilet facility (unimproved vs. improved)	9	1.13 (0.89, 1.45)	70.3	0.001	0.949
Water source (unimproved vs. improved)	12	1.05 (0.85, 1.30)	88.6	< 0.0001	0.947

a *Q* test for heterogeneity.

^b^ Funnel plot reported.

### 3.6 Pattern of Risk Factors Across Outcomes

A summary matrix of statistically significant associations across the three forms of undernutrition (stunting, underweight and wasting) including overall undernutrition is provided in Table S1. This table highlights common determinants such as diarrhoea, low birthweight and low maternal education, which were consistently associated with increased risk across multiple outcomes.

### 3.7 Heterogeneity and Risk of Publication Bias in Studies

Tables 2, 3, 4, 5 present the heterogeneity and potential publication bias across the included studies. Most analyses revealed substantial heterogeneity (*I*² > 50%), likely stemming from variations in how key variables were defined. For example, the definition for ‘high family size’ varied considerably – some studies classified it as more than two children, while others used a threshold of two or more, among other criteria. These inconsistencies prevented the use of a single standardised definition across studies.

Regarding publication bias, approximately 32% (*n* = 5) of the studies showed potential publication bias. The highest risk was observed for overall undernutrition and stunting, with 43% (*n* = 6) of studies in each category showing signs of publication bias. This was followed by underweight (29%, *n* = 4) and wasting (14%, *n* = 2).

### 3.8 Study Quality Assessment

Shown in Table 1 and Table S2 are the overall quality scores, and detail information of the quality scores, respectively. The median total score of the included studies was six (interquartile range: 5–7), indicating satisfactory methodological quality. However, certain methodological limitations were identified. Seven studies did not provide sufficient details about the study participants and setting, while one study failed to identify and adjust for potential confounders, as evidenced by the reporting of unadjusted or crude OR. In addition, more than half of the studies (*n* = 37) did not specify how confounding variables were addressed, though most did report crude or adjusted ORs.

## 4 Discussion

### 4.1 Summary of Findings

This systematic review and meta‐analysis examined the association between selected child‐related, household and hygiene and sanitation factors undernutrition including stunting, underweight and wasting among infants and young children aged 0 to 59 months in Sub‐Saharan Africa. Many of the risk factors identified coexist and collectively contributed to undernutrition outcomes. Notably, diarrhoea, low birthweight and low maternal education were consistently associated with overall undernutrition, stunting, underweight and wasting.

### 4.2 Immediate Factors of Undernutrition

Diarrhoea as a major immediate factor, showing significant associations across all outcomes. Although preventable and treatable, diarrhoea remains a leading cause of morbidity and mortality among young children (WHO 2024a). It leads to nutrient loss and dehydration, both critical factors for growth faltering (WHO 2024a). Our findings align with a study in three Sub‐Saharan African countries, where diarrhoea increased the risk of stunting by 30% (Nasrin et al. 2023).

Low birthweight was another significant immediate factor, consistently associated with undernutrition including stunting, underweight and wasting. Infants born with low birthweight have immature immune systems and poor nutritional reserves, making them highly vulnerable to infections (Jana et al. 2023; Kapti et al. 2022). An estimated 18.5 million low birthweight infants are born annually in low‐ and middle‐income countries (UNICEF 2023). Survivors are at increased risk of long‐term adverse outcomes, including growth retardation, cognitive impairment and non‐communicable diseases in adulthood (Abbas et al. 2021; Jornayvaz et al. 2016). Our meta‐analysis confirms the association between low birthweight and overall undernutrition, stunting, underweight and wasting, consistent with studies in Africa and Asia, where undernutrition prevalence is highest (Abbas et al. 2021; Jana et al. 2023; Namiiro et al. 2023; Ntenda 2019). Socioeconomic status and low maternal education have also been reported as contributing factors to low birthweight (Diabelková et al. 2022; Rahman et al. 2016).

### 4.3 Underlying Factors of Undernutrition

Poor hygiene and sanitation are crucial contributors to the undernutrition‐infection cycle. In our analysis, diarrhoea was consistently associated with overall undernutrition, stunting, underweight and wasting, while lack of handwashing was linked to overall undernutrition, underweight and wasting. These findings align with previous research showing that poor hygiene practices and inadequate safe water access exacerbate diarrhoeal disease risk and subsequent nutritional deficits (Sahiledengle et al. 2024; WHO 2024a). Moreover, children from large households often with multiple young children may face resource constraints that further limit adequate hygiene and care (Ahmad et al. 2020; Nasrin et al. 2023).

Low maternal education emerged as another significant underlying factor. Children of mothers with lower education levels had a higher likelihood of experiencing undernutrition, including stunting, underweight and wasting. Yorke et al. (2023) analysed Demographic and Health Surveys data from three West African countries and found that children of mothers with only primary education or less were at higher risk of stunting, underweight and wasting than those with secondary education or higher. A systematic review confirmed an inverse relationship between maternal education and childhood undernutrition, particularly in middle‐income countries and low‐education populations (Rezaeizadeh et al. 2024). This may be because educated mothers are more likely to make informed decisions regarding nutrition, healthcare, hygiene and timely medical interventions for their children (Ahmad et al. 2020; Kavosi et al. 2014).

### 4.4 Basic Factors of Undernutrition

Socioeconomic status plays a crucial role in childhood undernutrition. Our study found that children from low‐income households and rural communities were at a higher risk of stunting and wasting. This aligns with findings from Sub‐Saharan Africa, where undernutrition including stunting, underweight and wasting is more prevalent among children from poor households, particularly in rural areas (Alaba et al. 2023). Economic constraints limit access to nutrient‐rich foods, healthcare services, safe water and sanitation facilities (Ahmad et al. 2020). Furthermore, rural areas often lack adequate health services and education opportunities, exacerbating the problem (Srinivasan et al. 2013).

### 4.5 Sources of Heterogeneity

While our meta‐analysis accounted for between‐study heterogeneity using appropriate statistical methods, variations in study characteristics contributed to observed heterogeneity.

Differences in study designs (e.g., cross‐sectional vs. case‐control studies), variations in exposure and outcome definitions and inconsistencies in confounder adjustments might have influenced heterogeneity. Some studies categorised risk factors differently, such as food insecurity or maternal education, leading to a likely difference in the observed associations. Population differences, including socioeconomic conditions, healthcare access and cultural feeding practices, might have further contributed to heterogeneity. For example, the impact of maternal education on childhood nutrition may vary based on local policies and economic stability.

A notable source of heterogeneity was the geographic concentration of studies. A significant proportion (30 out of 49) were conducted in Ethiopia, which might have limited the generalisability of findings to the broader Sub‐Saharan African region. Ethiopia's unique sociocultural and economic conditions may impact undernutrition differently than other countries. Future research should aim for greater geographical diversity to improve representativeness.

To address these variations, we conducted subgroup and sensitivity analyses to explore the impact of study design, geographic region and methodological approaches. However, some degree of heterogeneity remains unavoidable. These considerations also inform the broader limitations of the study.

### 4.6 Limitations

In addition to the heterogeneity discussed above, this study has several other limitations. First, most included studies were cross‐sectional, limiting causal inference. Second, the majority of studies were conducted in Ethiopia, necessitating research in other Sub‐Saharan African countries to enhance generalisability. Third, the study focused on selected risk factors, potentially overlooking other key determinants of undernutrition. Fourth, inconsistency in variable definitions, such as family size may have introduced variability in findings. Standardisation in future research would improve comparability. Fifth, many studies lacked clarity on confounder adjustments, raising the possibility of residual confounding bias. Finally, the assessment of publication bias was limited by the small number of studies available for some risk factors. Although funnel plots were used for visual inspection, their interpretability is restricted when few studies are included, and statistical tests for publication bias are not reliable in such cases.

### 4.7 Strengths of the Study

This study has several strengths. First, rigorous statistical methods were used to enhance reliability. Second, the inclusion of a large number of studies increased statistical power. Third, analysing multiple exposure factors provided a comprehensive assessment. Finally, many included studies accounted for confounders, improving result accuracy.

## 5 Conclusion

This systematic review and meta‐analysis provide strong evidence that diarrhoea, low birthweight and low maternal education are significant risk factors for undernutrition including stunting, underweight and wasting among infants and young children in Sub‐Saharan Africa. These findings highlight the need for targeted interventions, including: (1) Improved hygiene and sanitation practices to reduce diarrhoea‐related undernutrition, (2) programmes supporting maternal education, particularly in low‐income communities, and (3) policies addressing food security and healthcare accessibility to improve child nutrition outcomes. Future research should adopt prospective study designs to validate findings and ensure better standardisation of variable definitions for cross‐study comparisons.

## Author Contributions

H.R. conceptualised the study. H.R. and C.R. designed the study. C.R. wrote the analytical codes. H.R. collected, analysed, and interpreted the data, as well as drafted the first version of the manuscript. C.R. supervised the data analysis and the drafting of the manuscript. C.R., D.S., S.K., and S.T. proofread the manuscript. All authors approved the final version of the manuscript for submission.

## Conflicts of Interest

The authors declare no conflicts of interest.

## Supporting information

forest anaemic st.

forest anaemic all.

forest anaemic uw.

forest anaemic wt.

forest area of residence all.

forest area of residence st.

forest area of residence uw.

forest area of residence wt.

forest BF all.

forest BF st.

forest BF uw.

forest BF wt.

forest BW all.

forest BW st.

forest BW uw.

forest BW wt.

forest diarrhoea all.

forest diarrhoea st.

forest diarrhoea uw.

forest diarrhoea wt.

forest family size all.

forest family size st.

forest family size uw.

forest family size wt.

forest food security all.

forest food security st.

forest food security uw.

forest food security wt.

forest handwashing all.

forest handwashing st.

forest handwashing uw.

forest handwashing wt.

forest maternal edu all.

forest maternal edu st.

forest maternal edu uw.

forest maternal edu wt.

forest no of children all.

forest no of children st.

forest no of children uw.

forest no of children wt.

forest SES all.

forest SES st.

forest SES uw.

forest SES wt.

forest suppl wt.

forest suppl all.

forest suppl st.

forest suppl uw.

forest toilet facility all.

forest toilet facility st.

forest toilet facility uw.

forest toilet facility wt.

forest water source all.

forest water source st.

forest water source uw.

forest water source wt.

Suppl table 1 pattern of risk factors fin 10062025.

Suppl table 2 quality scores.

Supplementary material 1 25042025.

## Data Availability

Data sharing available upon request.
