# Supplementary figures and images for: Factors Associated With Childhood Undernutrition in Sub‐Saharan Africa: A Systematic Review and Meta‐Analysis
Source: Matern Child Nutr. 2025 Aug 31;22(1):e70083. doi: 10.1111/mcn.70083 (PMC12893520; doi:10.1111/mcn.70083)

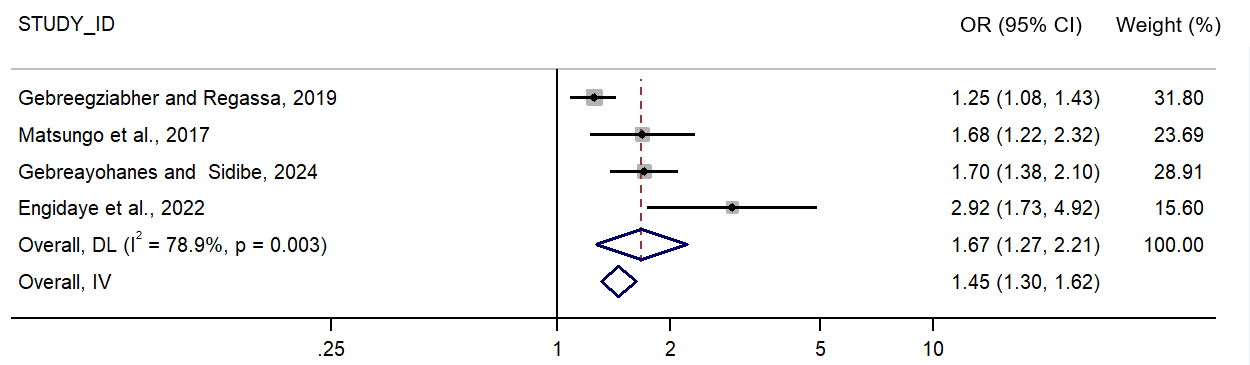

Supplement: Supplementary file 1 — forest anaemic st. [file MCN-22-e70083-s055.tif]

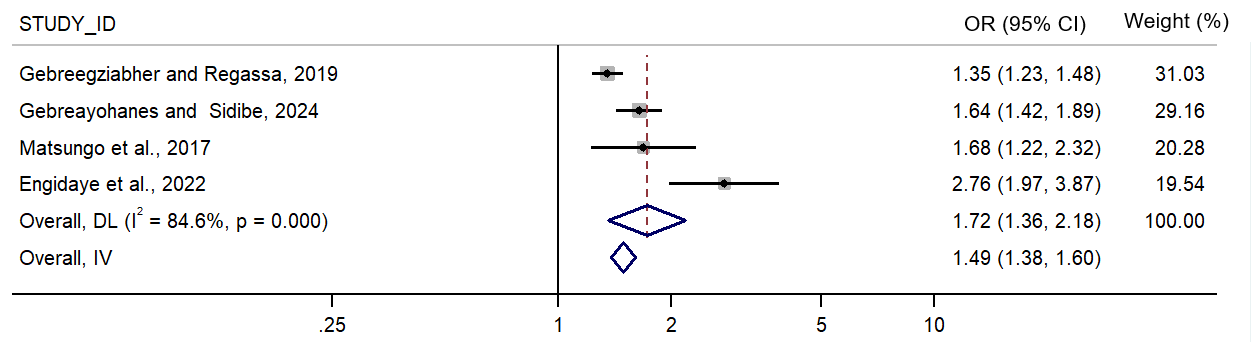

Supplement: Supplementary file 2 — forest anaemic all. [file MCN-22-e70083-s052.tif]

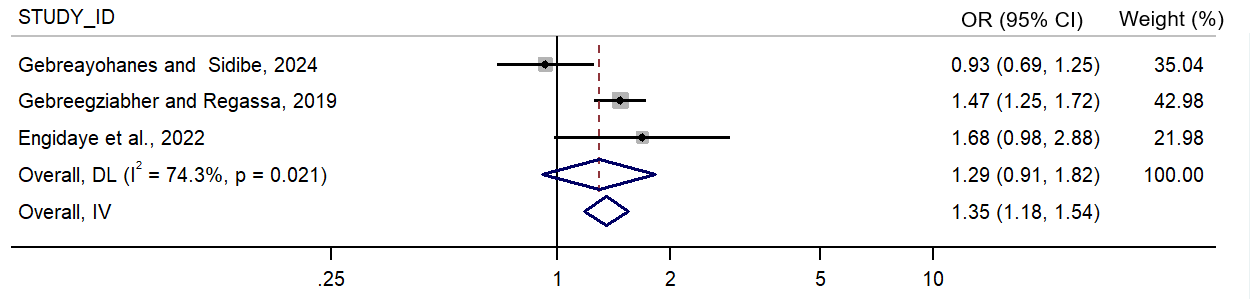

Supplement: Supplementary file 3 — forest anaemic uw. [file MCN-22-e70083-s022.tif]

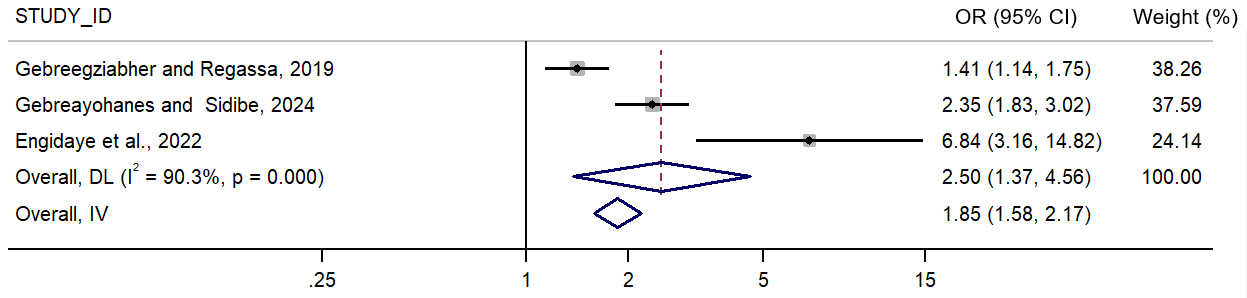

Supplement: Supplementary file 4 — forest anaemic wt. [file MCN-22-e70083-s056.tif]

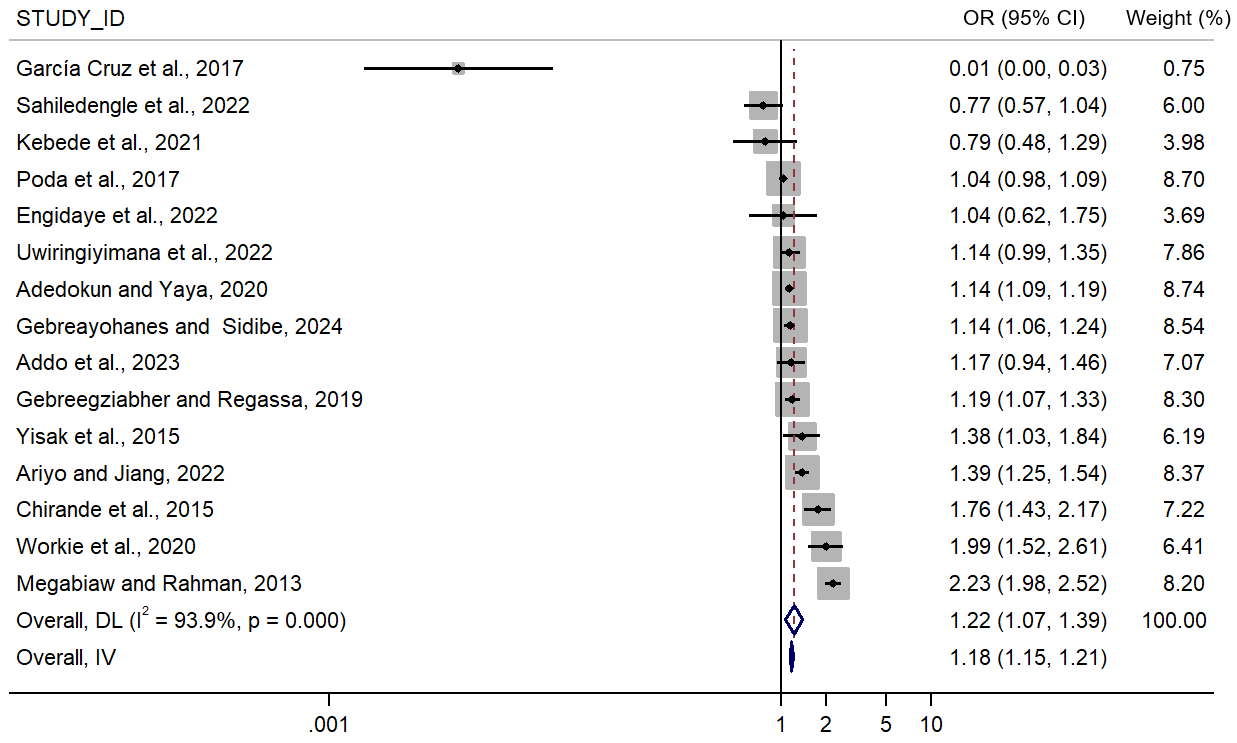

Supplement: Supplementary file 5 — forest area of residence all. [file MCN-22-e70083-s040.tif]

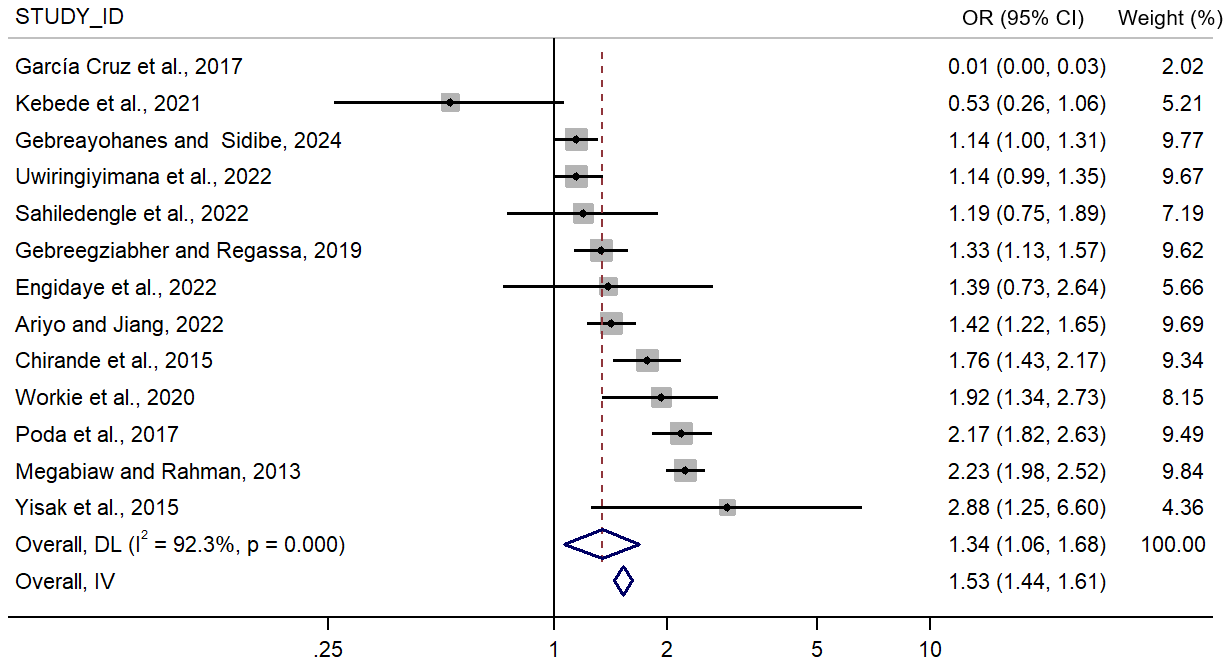

Supplement: Supplementary file 6 — forest area of residence st. [file MCN-22-e70083-s048.tif]

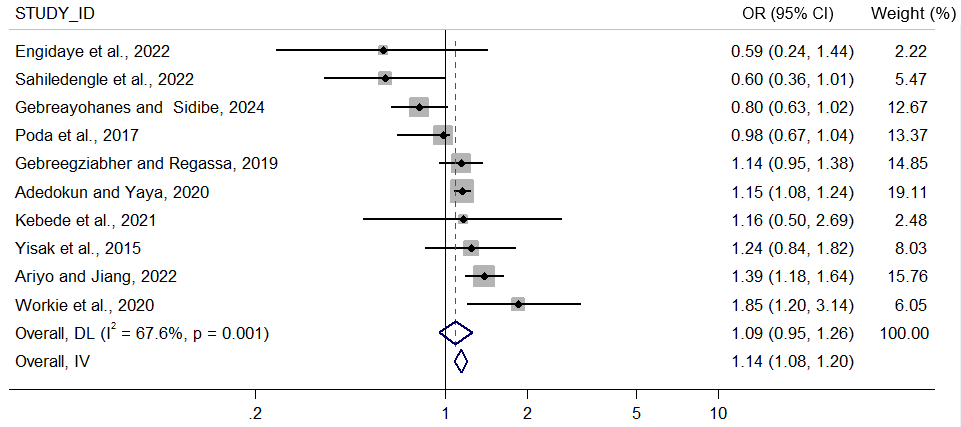

Supplement: Supplementary file 7 — forest area of residence uw. [file MCN-22-e70083-s019.tif]

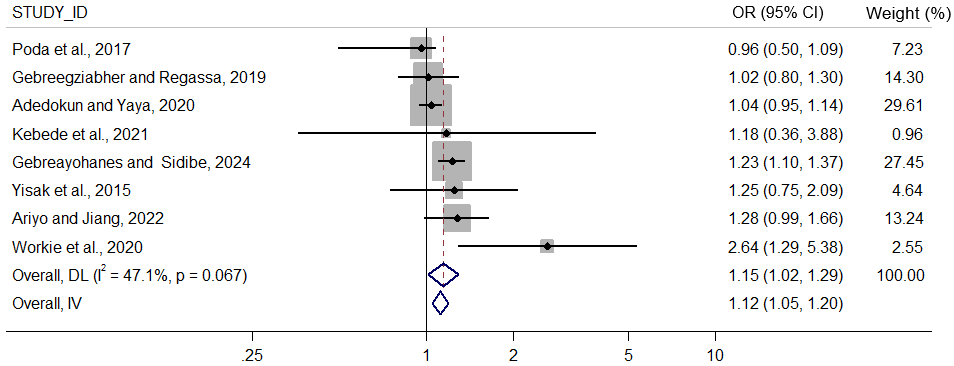

Supplement: Supplementary file 8 — forest area of residence wt. [file MCN-22-e70083-s037.tif]

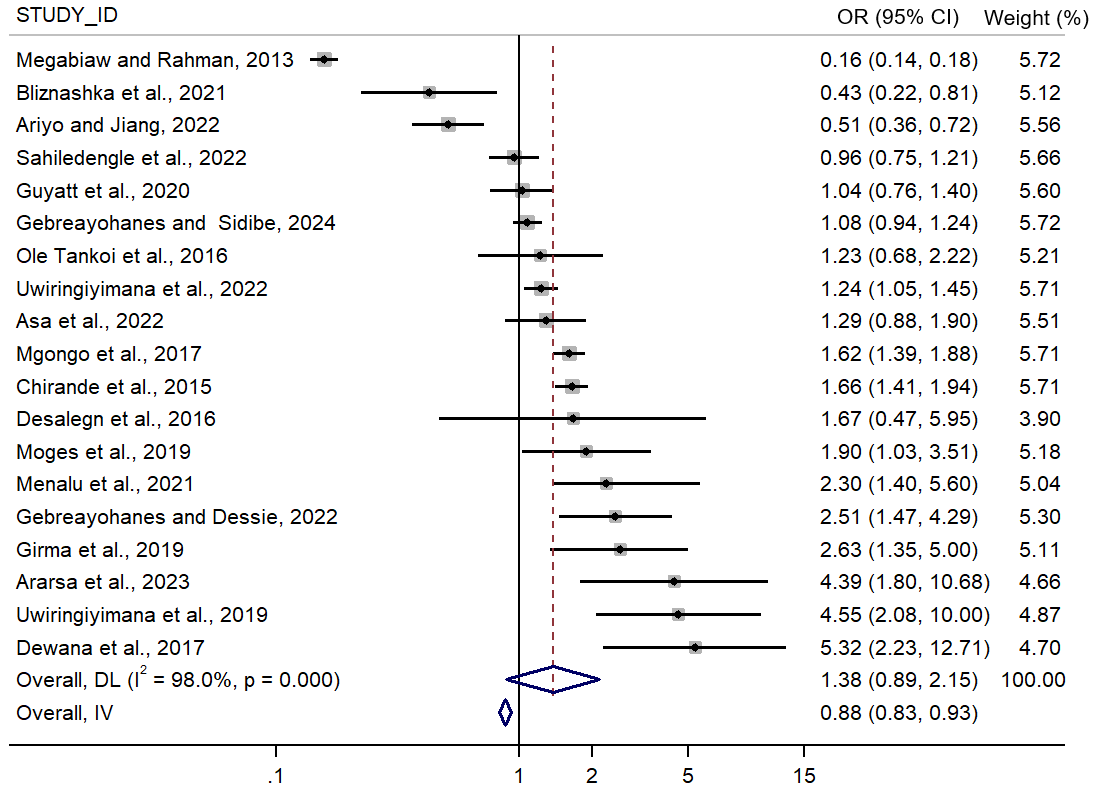

Supplement: Supplementary file 9 — forest BF all. [file MCN-22-e70083-s009.tif]

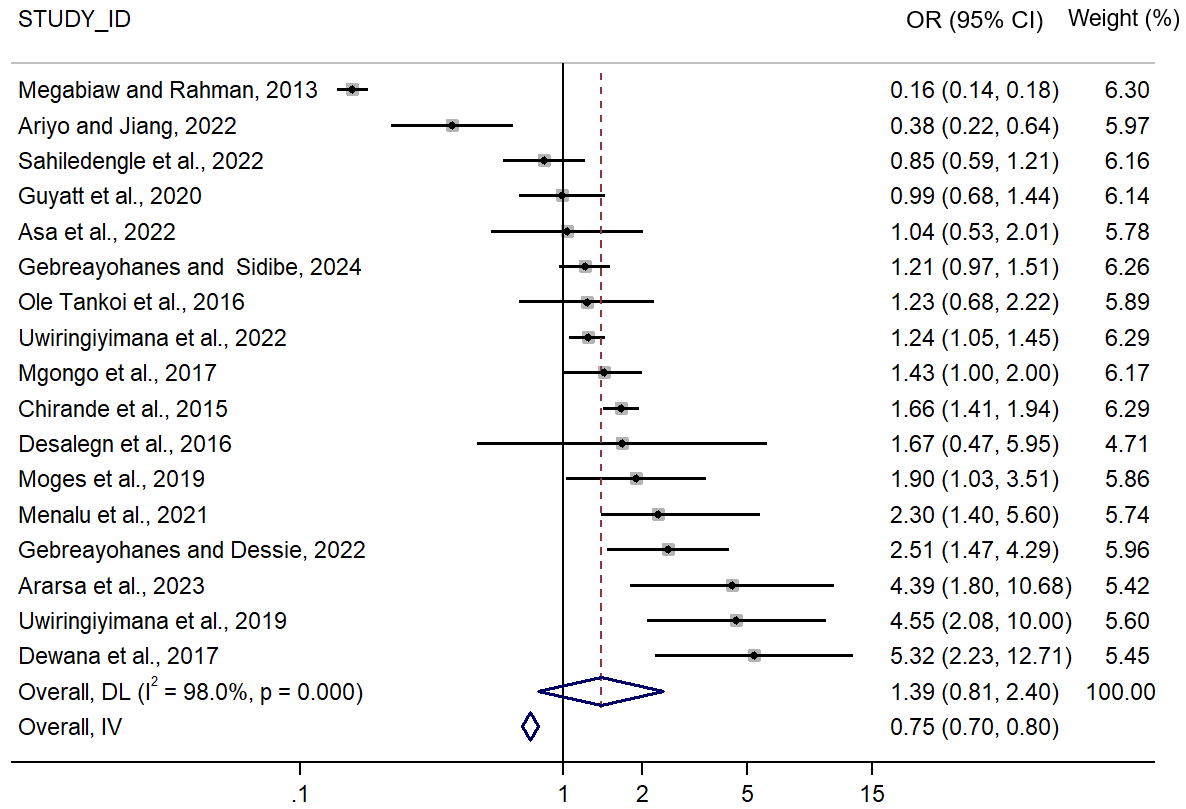

Supplement: Supplementary file 10 — forest BF st. [file MCN-22-e70083-s030.tif]

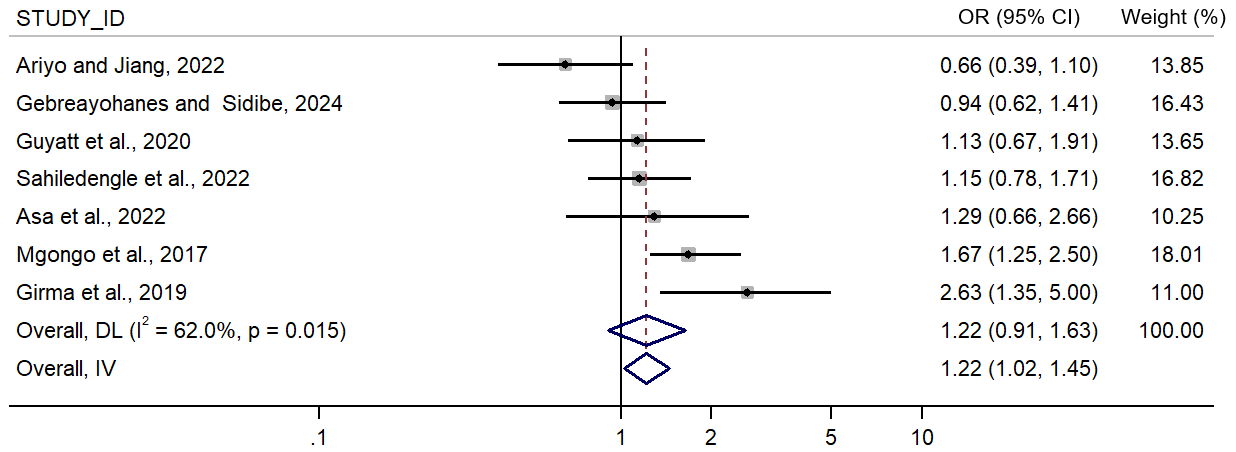

Supplement: Supplementary file 11 — forest BF uw. [file MCN-22-e70083-s046.tif]

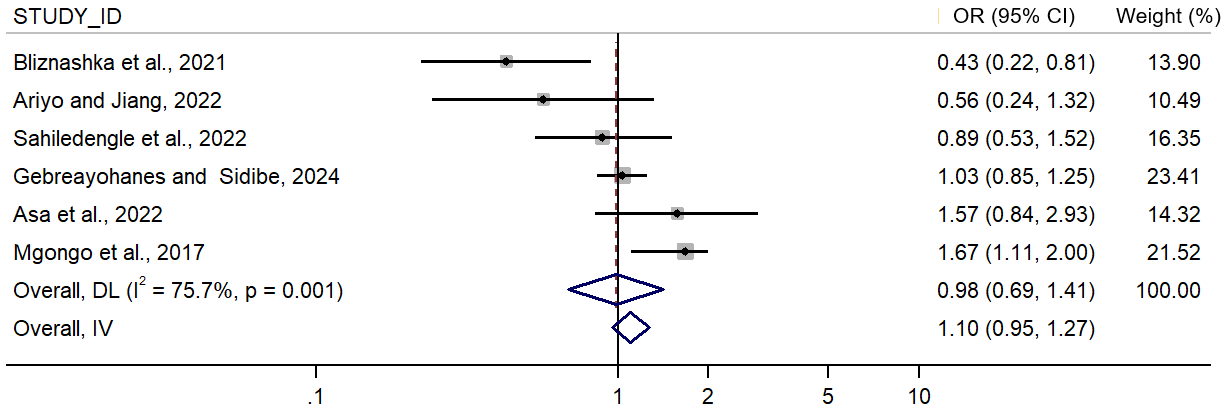

Supplement: Supplementary file 12 — forest BF wt. [file MCN-22-e70083-s053.tif]

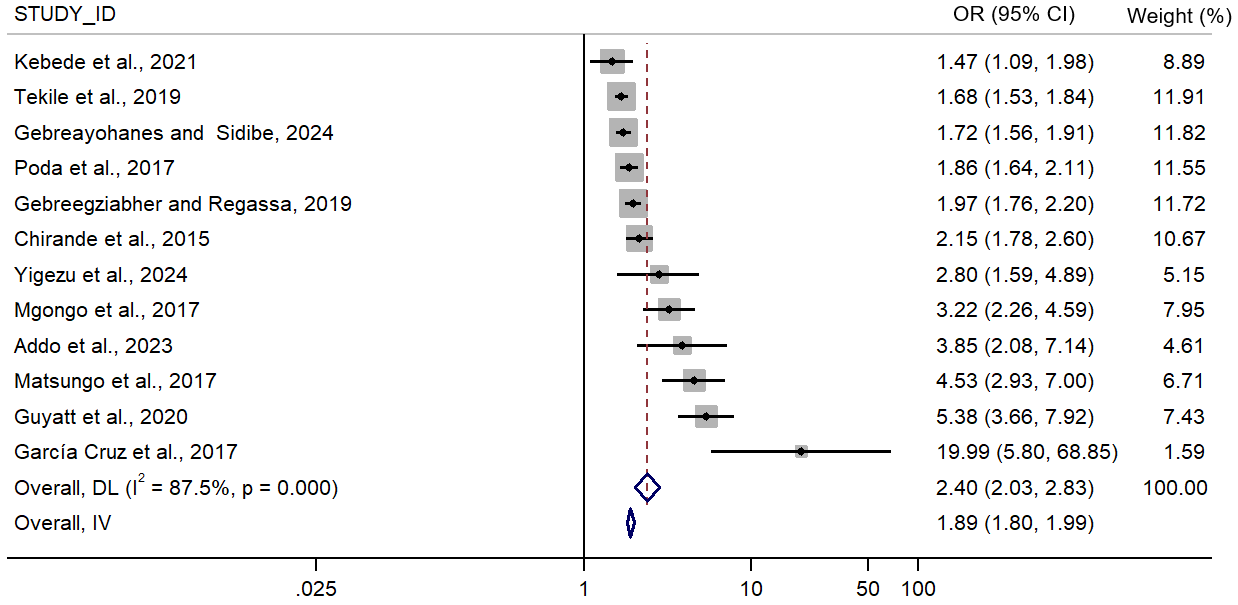

Supplement: Supplementary file 13 — forest BW all. [file MCN-22-e70083-s029.tif]

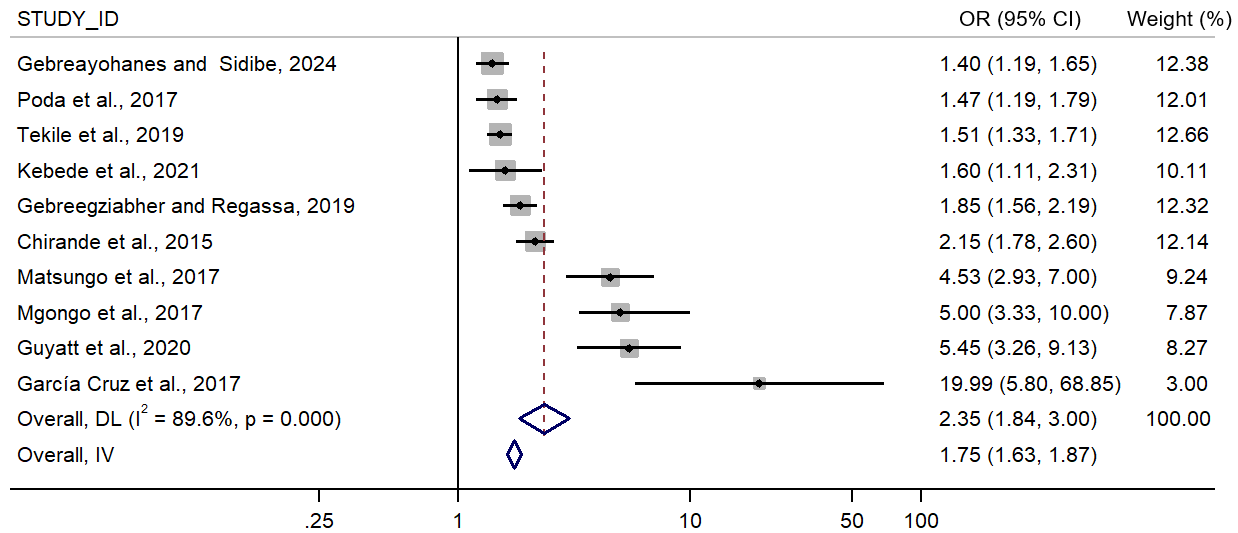

Supplement: Supplementary file 14 — forest BW st. [file MCN-22-e70083-s003.tif]

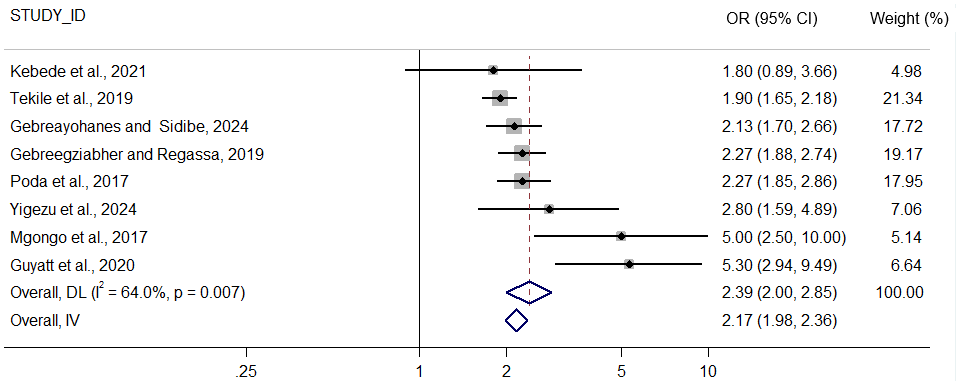

Supplement: Supplementary file 15 — forest BW uw. [file MCN-22-e70083-s018.tif]

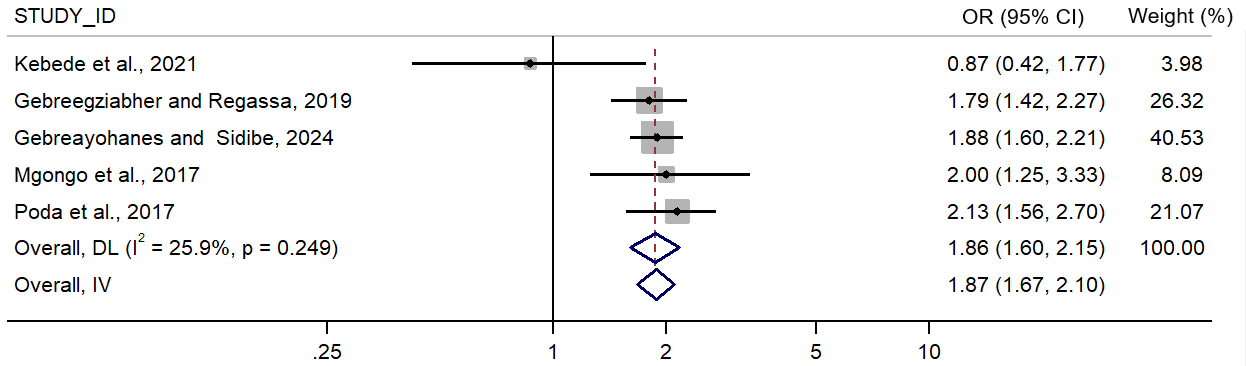

Supplement: Supplementary file 16 — forest BW wt. [file MCN-22-e70083-s038.tif]

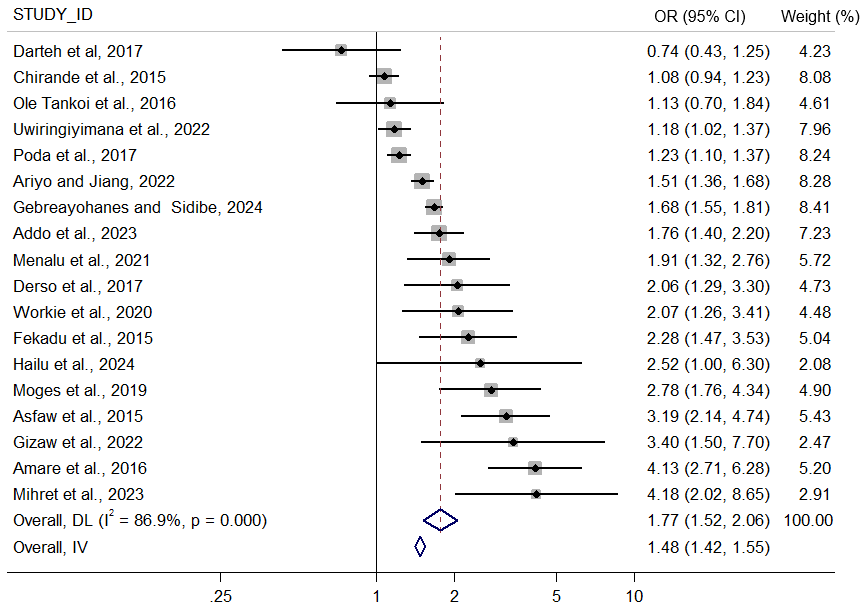

Supplement: Supplementary file 17 — forest diarrhoea all. [file MCN-22-e70083-s021.tif]

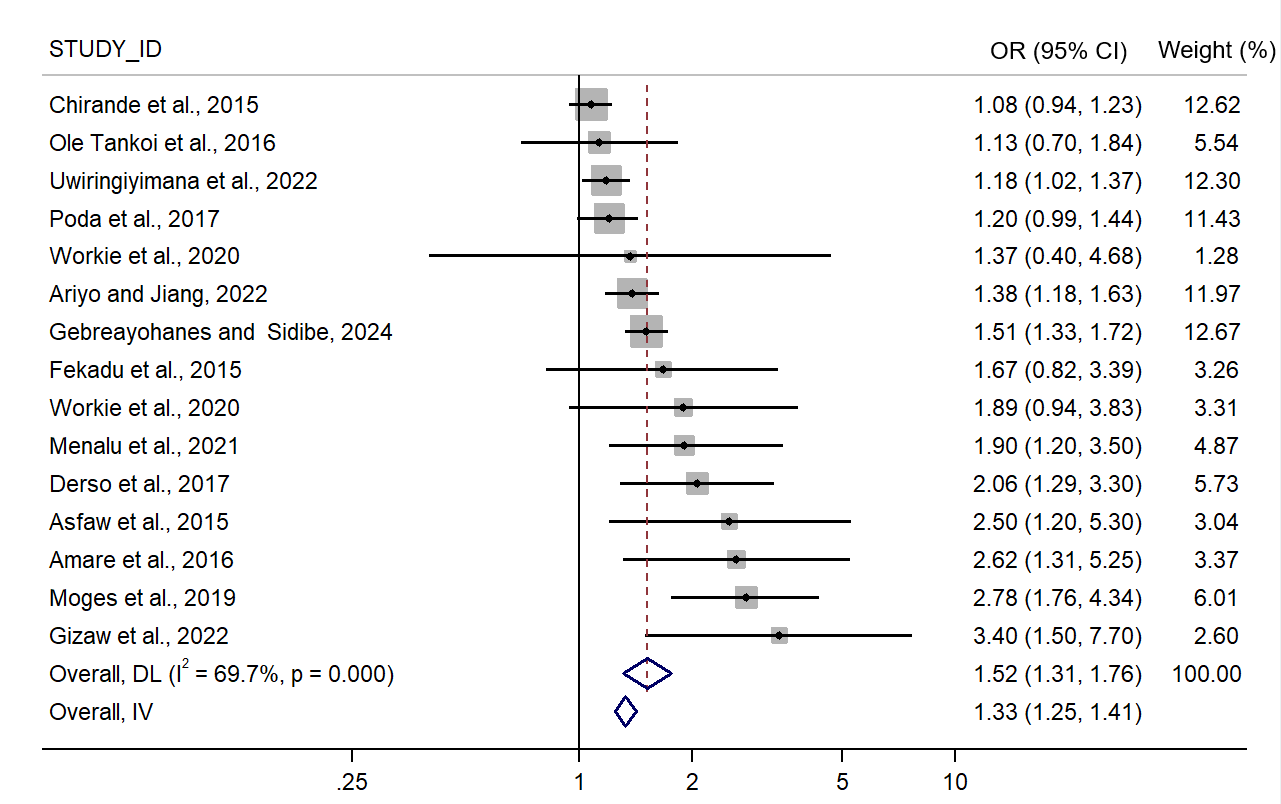

Supplement: Supplementary file 18 — forest diarrhoea st. [file MCN-22-e70083-s041.tif]

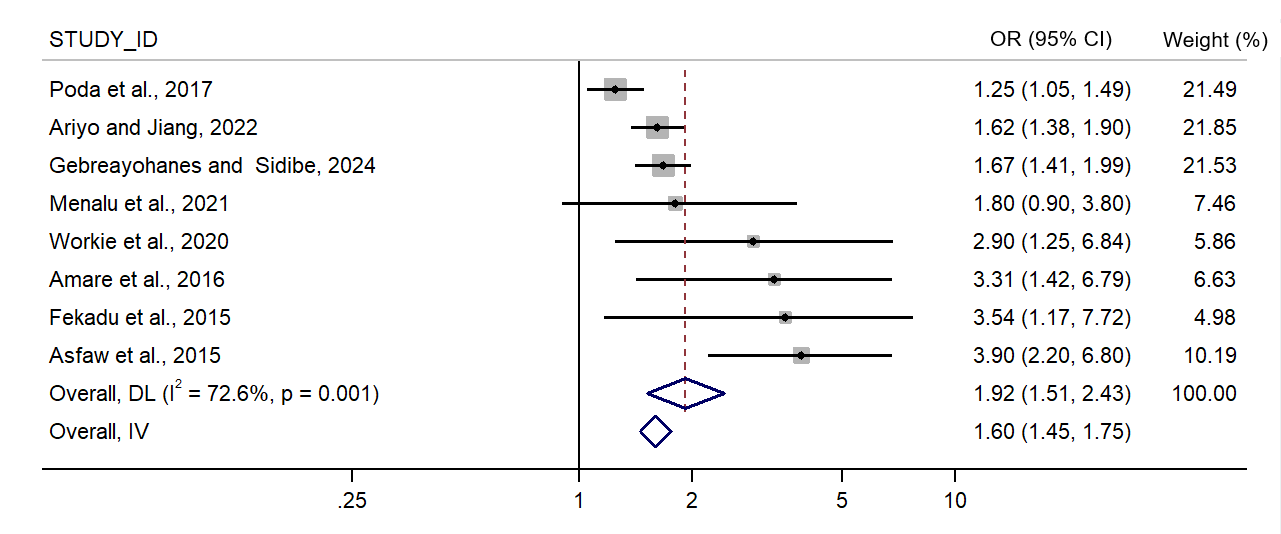

Supplement: Supplementary file 19 — forest diarrhoea uw. [file MCN-22-e70083-s044.tif]

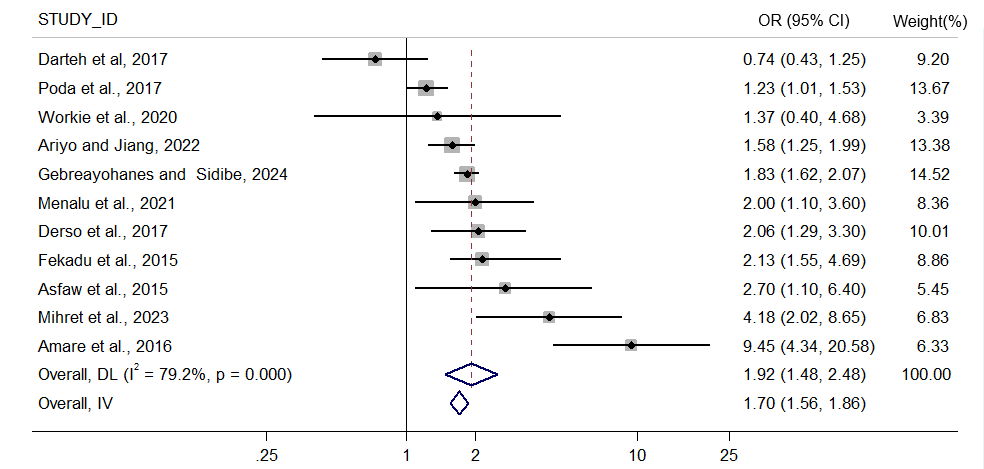

Supplement: Supplementary file 20 — forest diarrhoea wt. [file MCN-22-e70083-s031.tif]

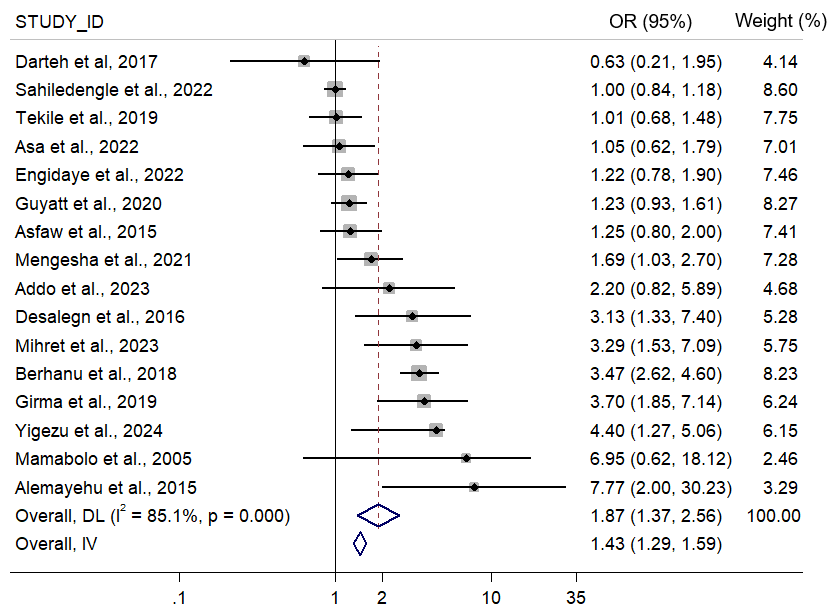

Supplement: Supplementary file 21 — forest family size all. [file MCN-22-e70083-s051.tif]

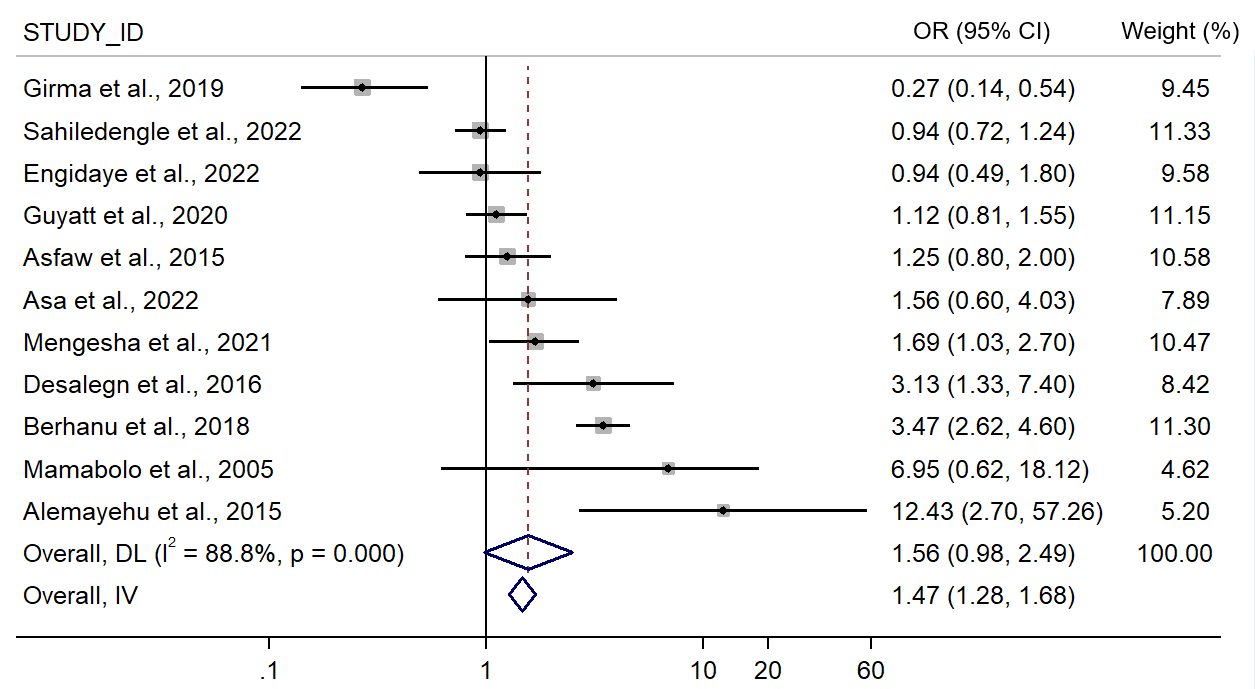

Supplement: Supplementary file 22 — forest family size st. [file MCN-22-e70083-s028.tif]

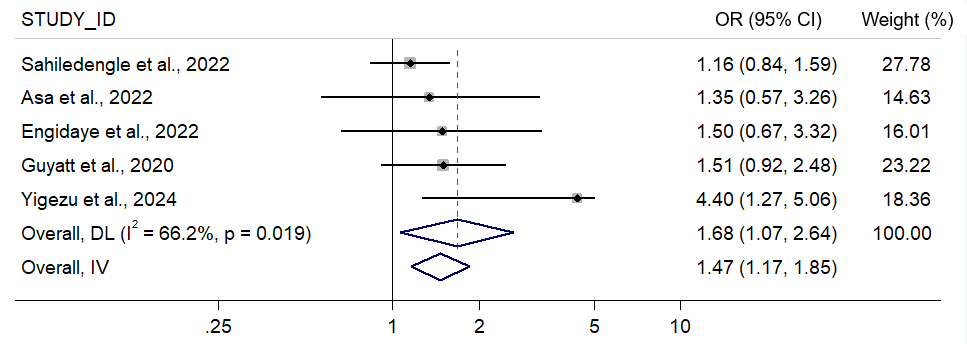

Supplement: Supplementary file 23 — forest family size uw. [file MCN-22-e70083-s023.tif]

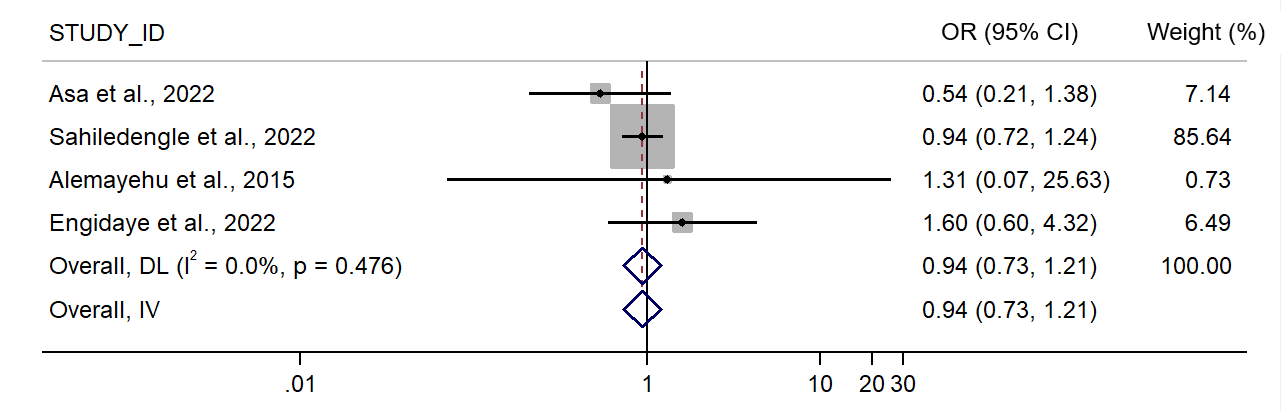

Supplement: Supplementary file 24 — forest family size wt. [file MCN-22-e70083-s036.tif]

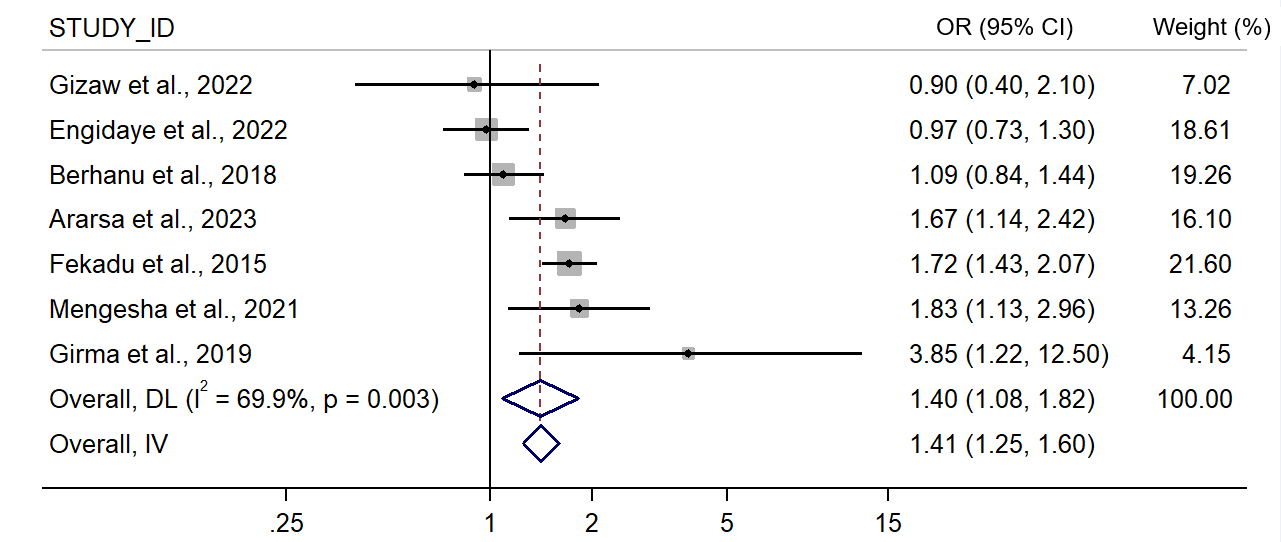

Supplement: Supplementary file 25 — forest food security all. [file MCN-22-e70083-s057.tif]

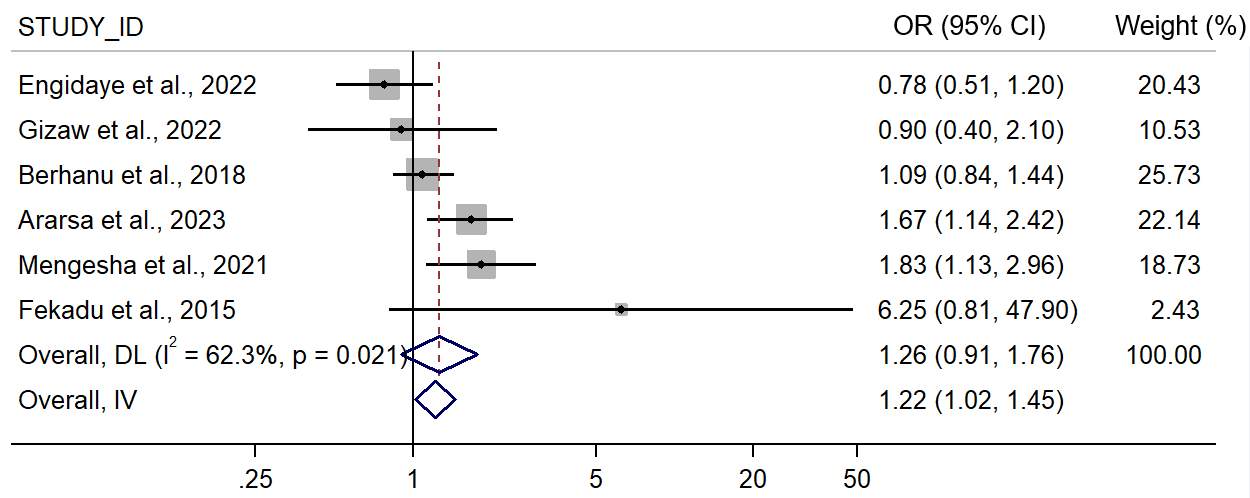

Supplement: Supplementary file 26 — forest food security st. [file MCN-22-e70083-s045.tif]

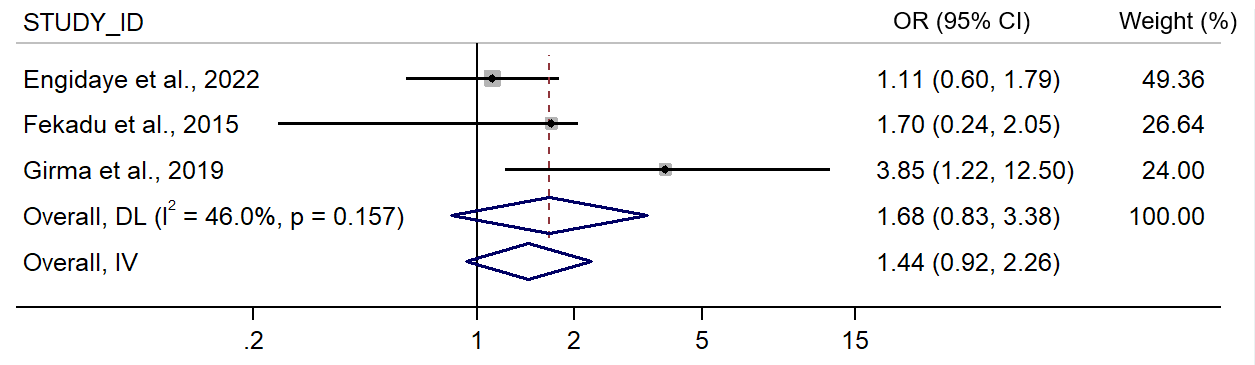

Supplement: Supplementary file 27 — forest food security uw. [file MCN-22-e70083-s059.tif]

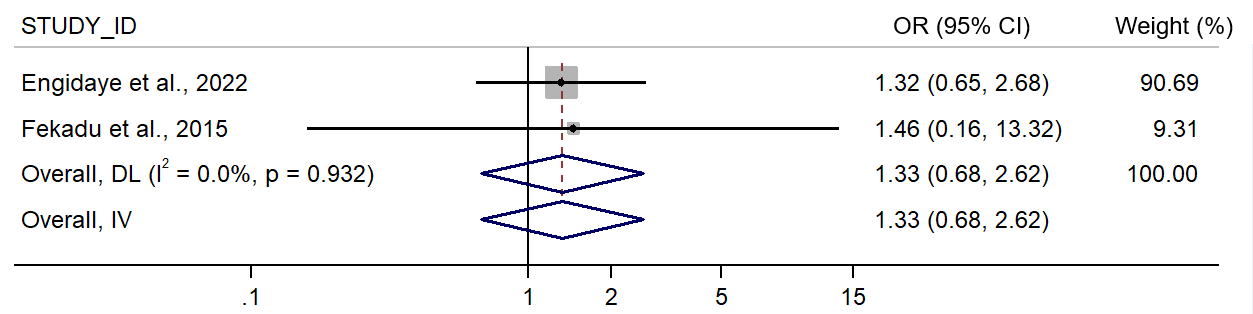

Supplement: Supplementary file 28 — forest food security wt. [file MCN-22-e70083-s013.tif]

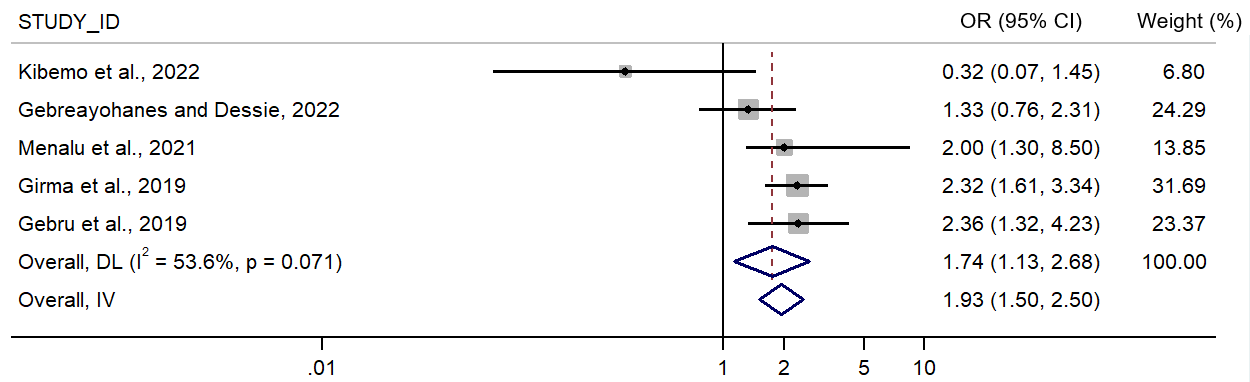

Supplement: Supplementary file 29 — forest handwashing all. [file MCN-22-e70083-s017.tif]

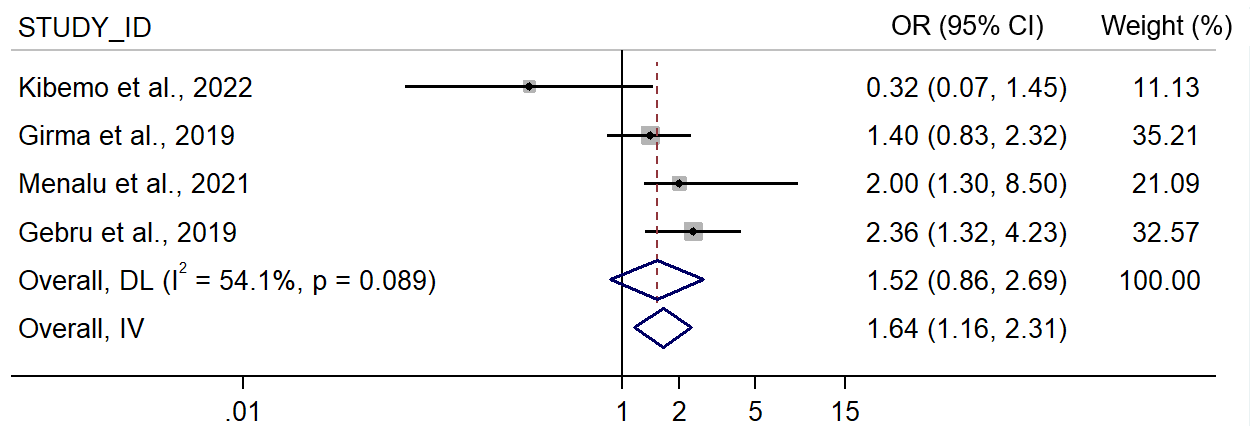

Supplement: Supplementary file 30 — forest handwashing st. [file MCN-22-e70083-s025.tif]

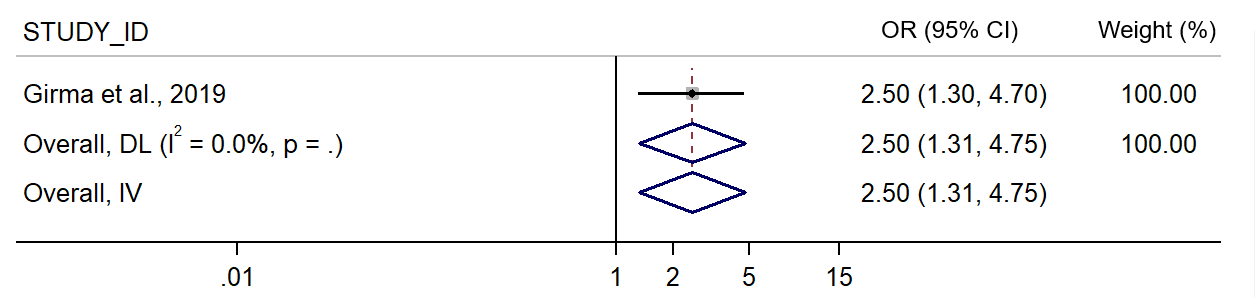

Supplement: Supplementary file 31 — forest handwashing uw. [file MCN-22-e70083-s026.tif]

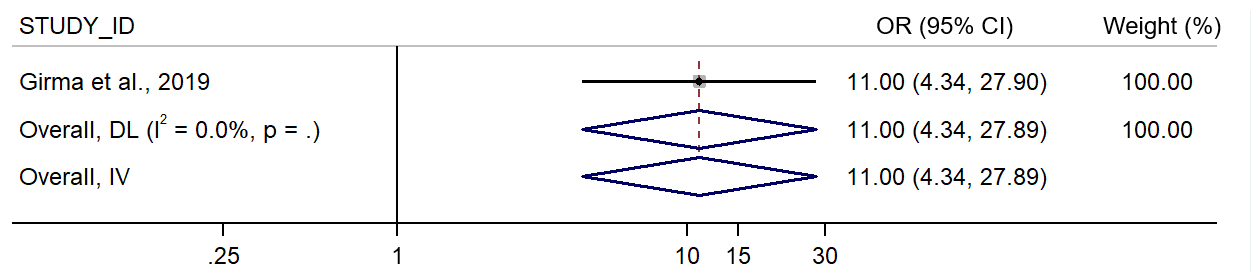

Supplement: Supplementary file 32 — forest handwashing wt. [file MCN-22-e70083-s011.tif]

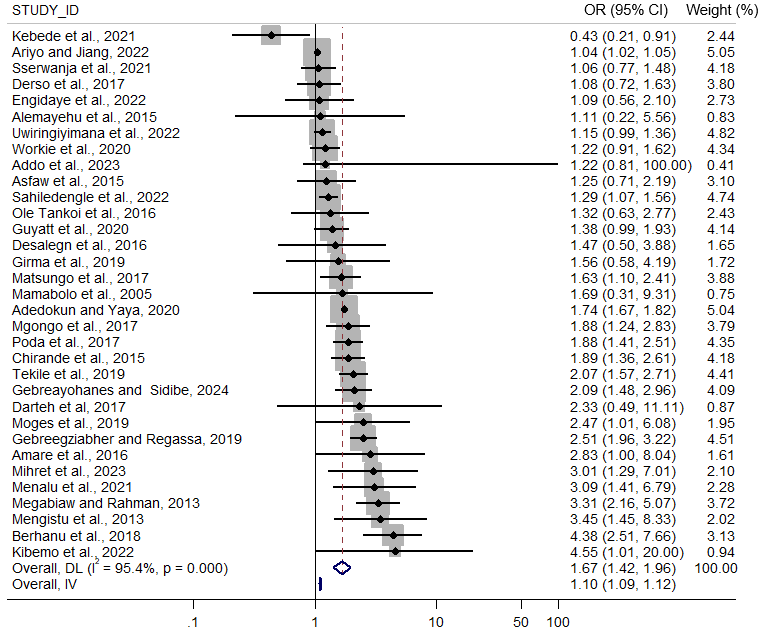

Supplement: Supplementary file 33 — forest maternal edu all. [file MCN-22-e70083-s024.tif]

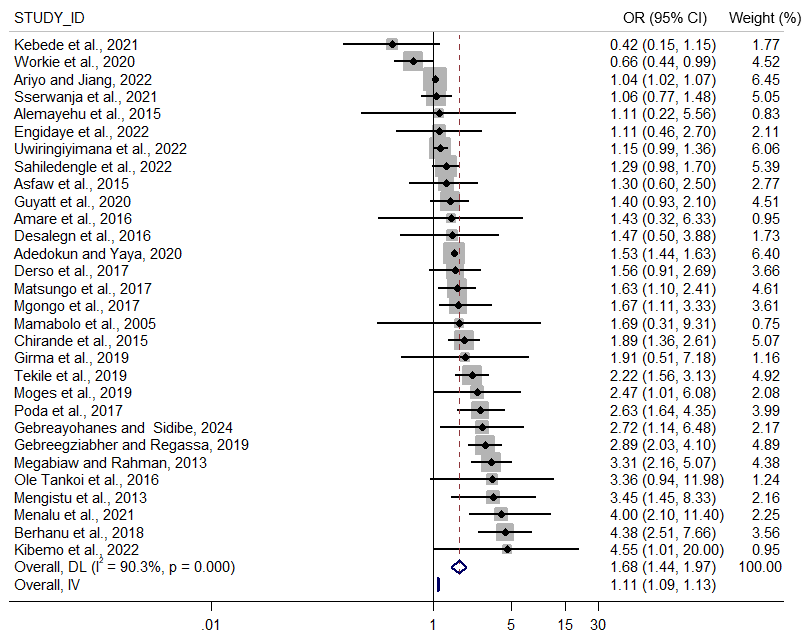

Supplement: Supplementary file 34 — forest maternal edu st. [file MCN-22-e70083-s054.tif]

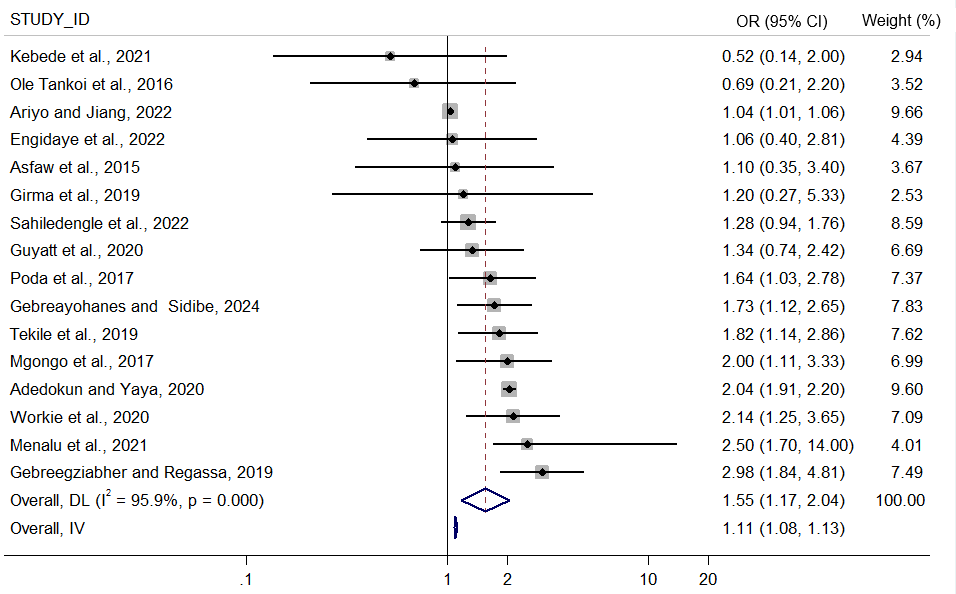

Supplement: Supplementary file 35 — forest maternal edu uw. [file MCN-22-e70083-s034.tif]

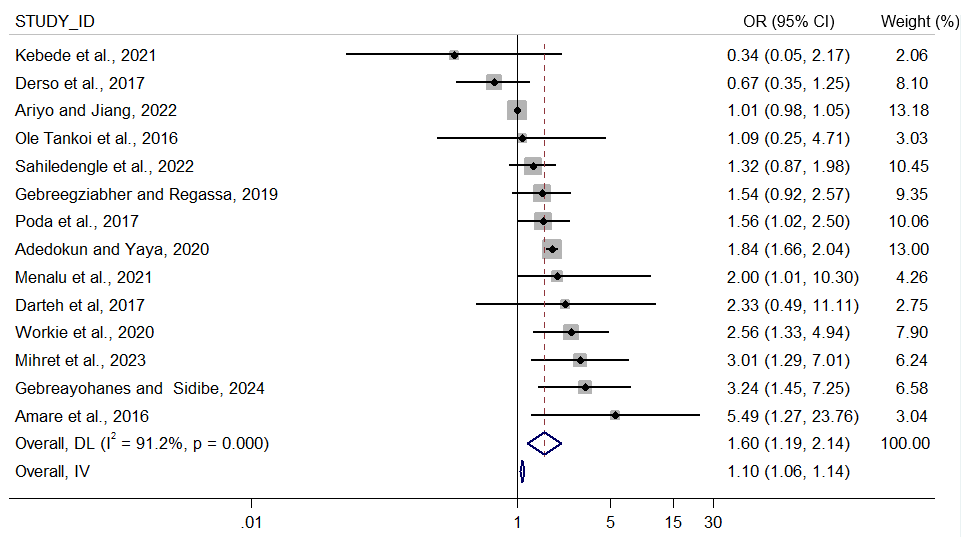

Supplement: Supplementary file 36 — forest maternal edu wt. [file MCN-22-e70083-s033.tif]

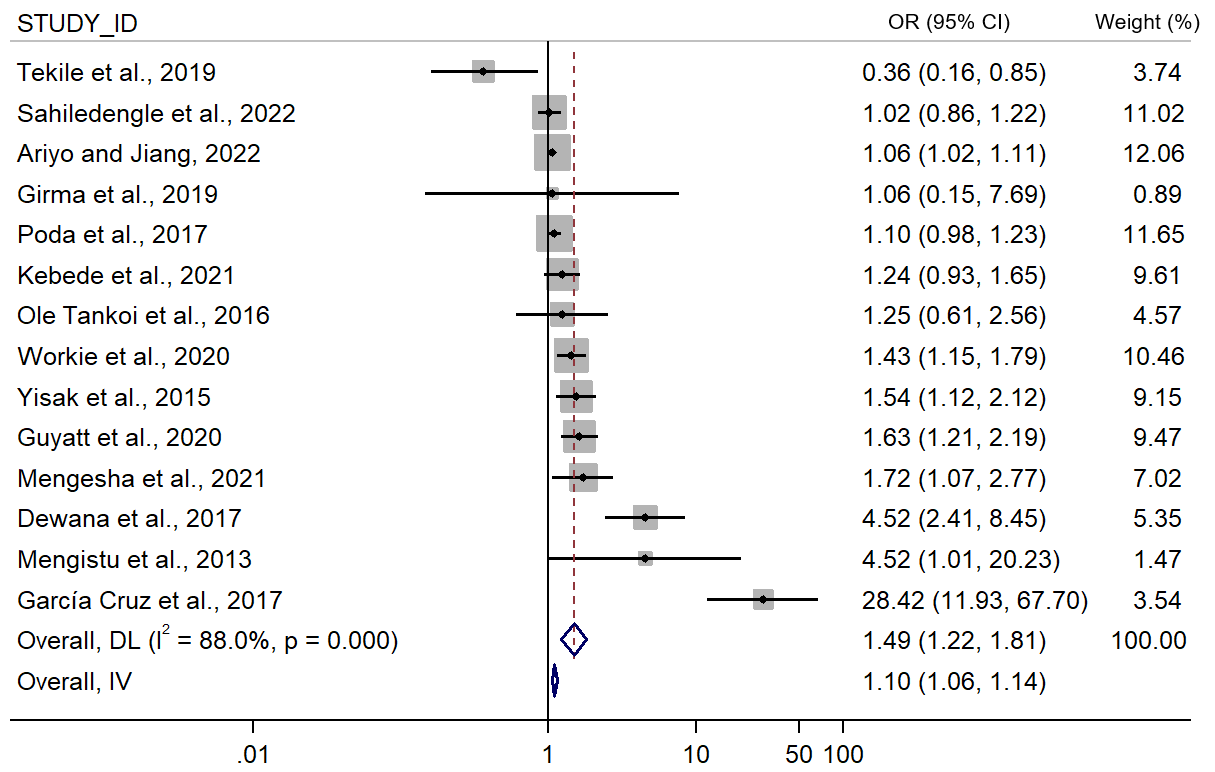

Supplement: Supplementary file 37 — forest no of children all. [file MCN-22-e70083-s047.tif]

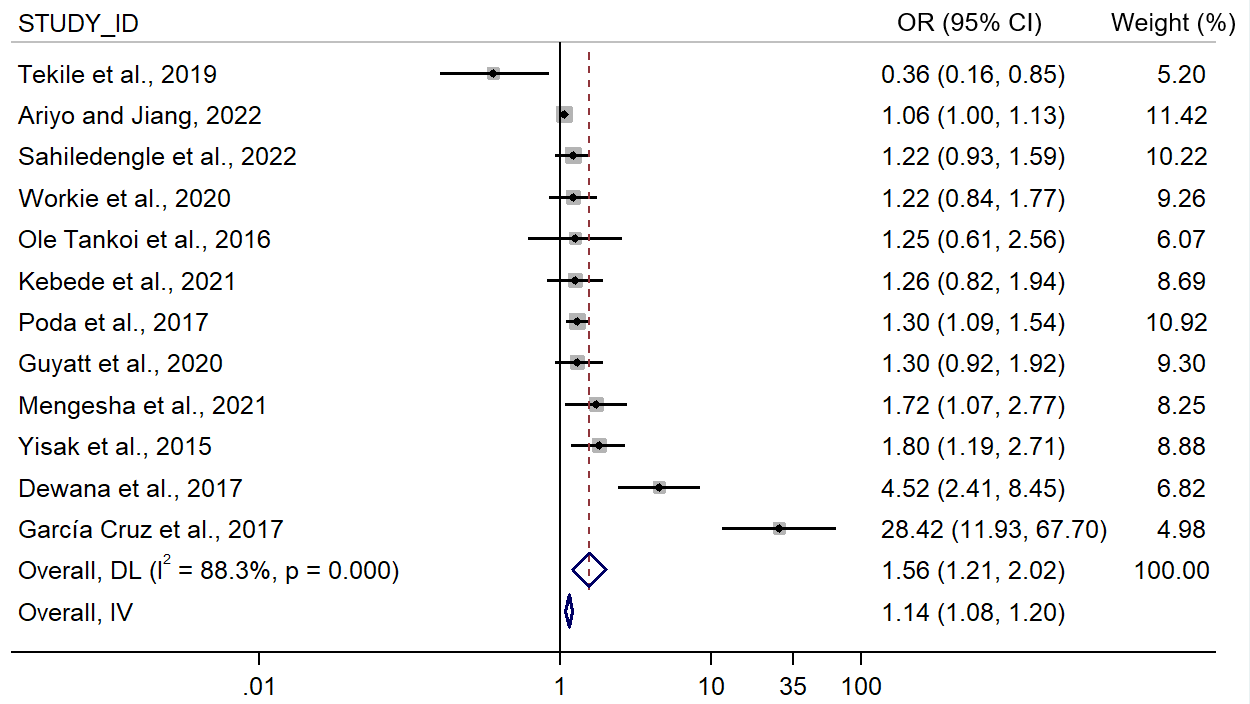

Supplement: Supplementary file 38 — forest no of children st. [file MCN-22-e70083-s005.tif]

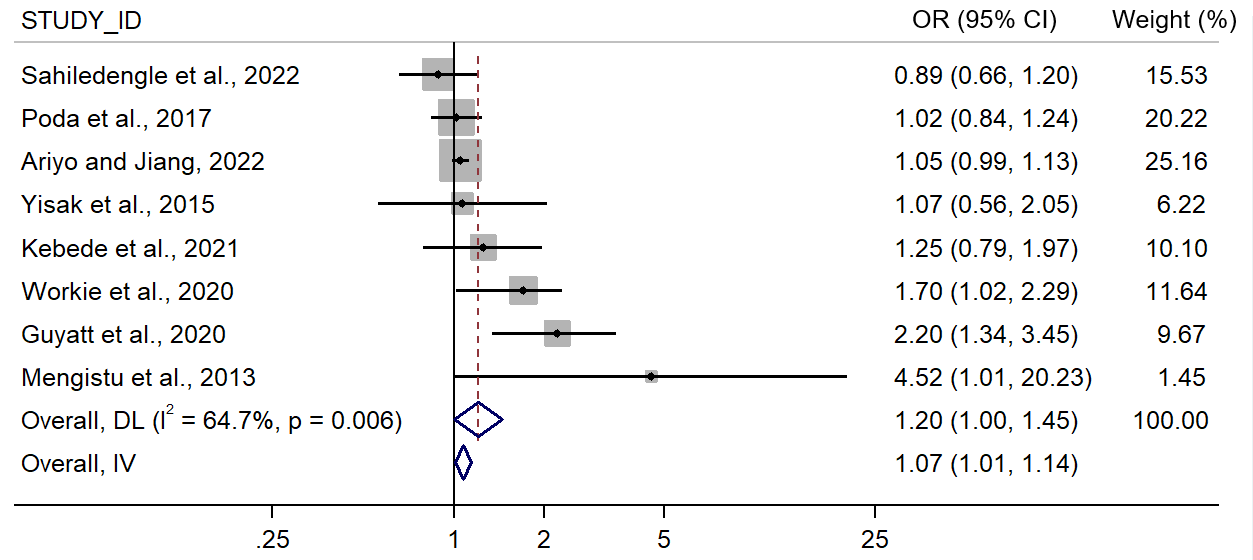

Supplement: Supplementary file 39 — forest no of children uw. [file MCN-22-e70083-s027.tif]

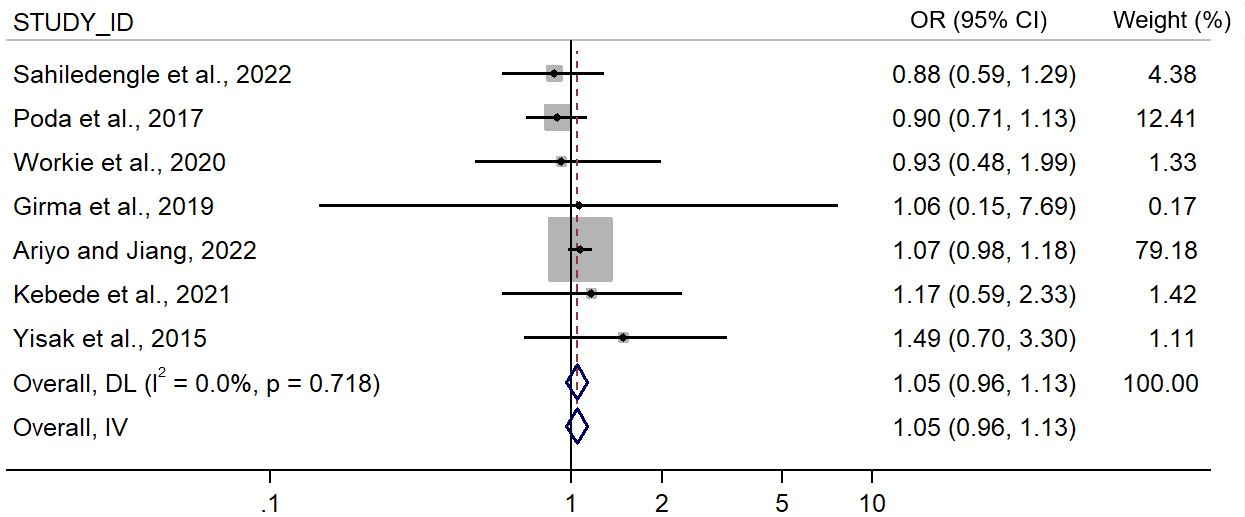

Supplement: Supplementary file 40 — forest no of children wt. [file MCN-22-e70083-s035.tif]

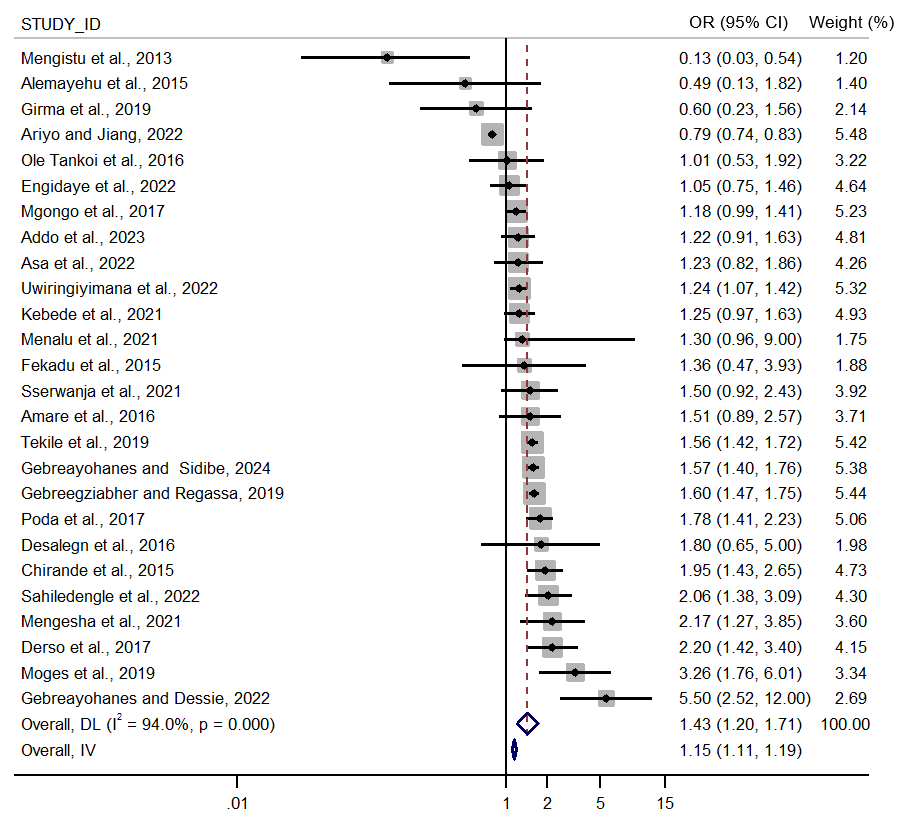

Supplement: Supplementary file 41 — forest SES all. [file MCN-22-e70083-s002.tif]

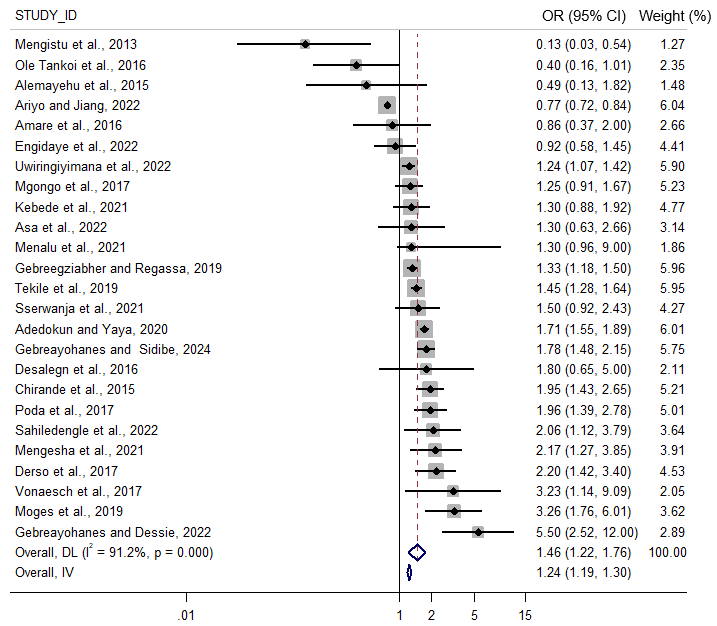

Supplement: Supplementary file 42 — forest SES st. [file MCN-22-e70083-s004.tif]

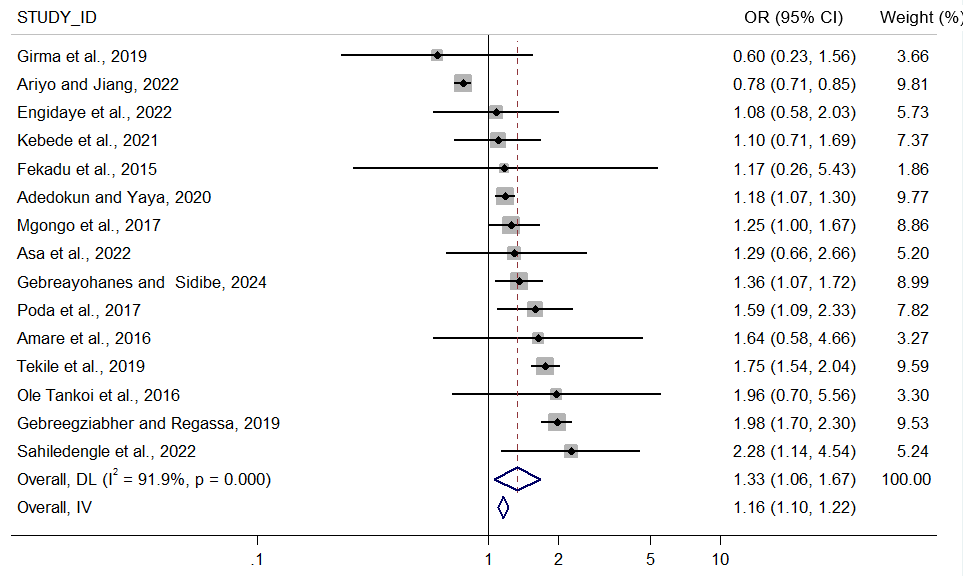

Supplement: Supplementary file 43 — forest SES uw. [file MCN-22-e70083-s020.tif]

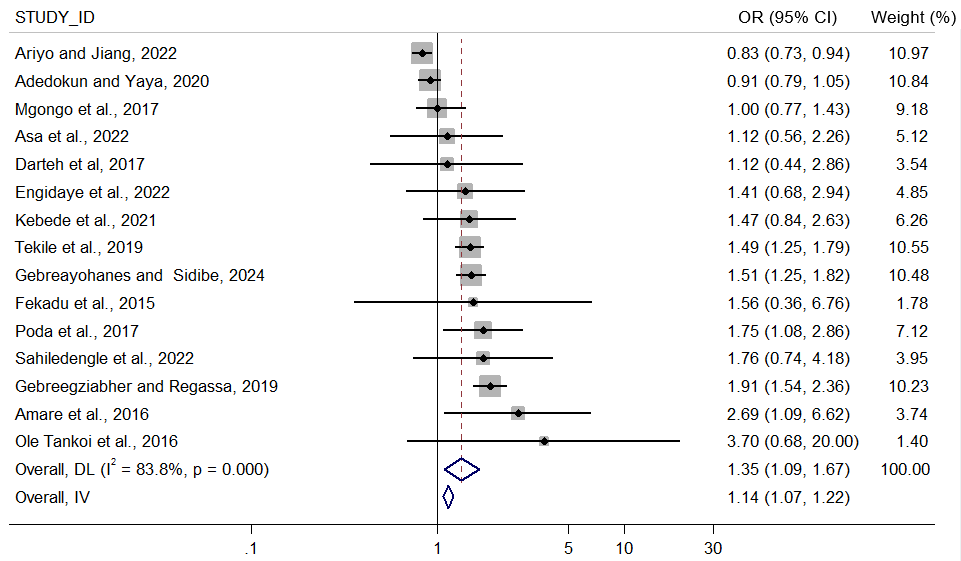

Supplement: Supplementary file 44 — forest SES wt. [file MCN-22-e70083-s008.tif]

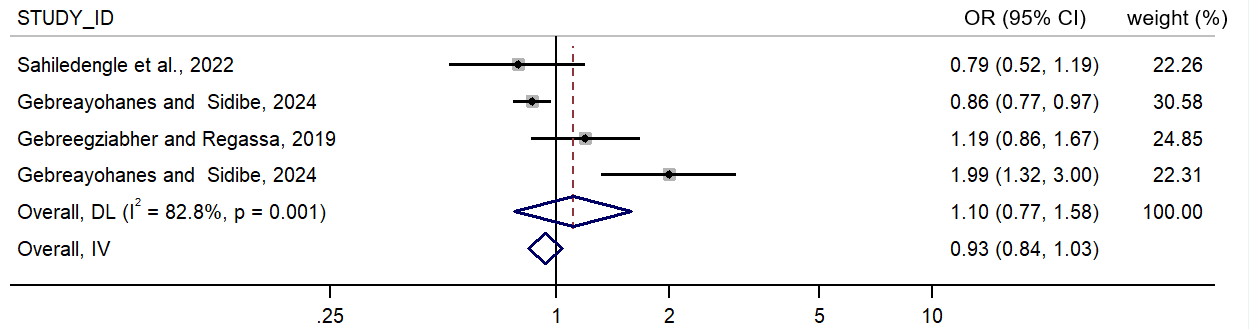

Supplement: Supplementary file 45 — forest suppl wt. [file MCN-22-e70083-s006.tif]

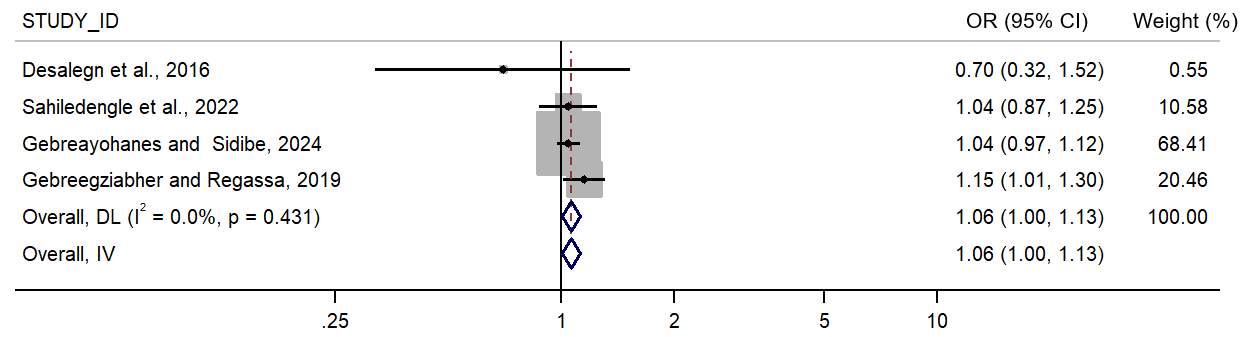

Supplement: Supplementary file 46 — forest suppl all. [file MCN-22-e70083-s042.tif]

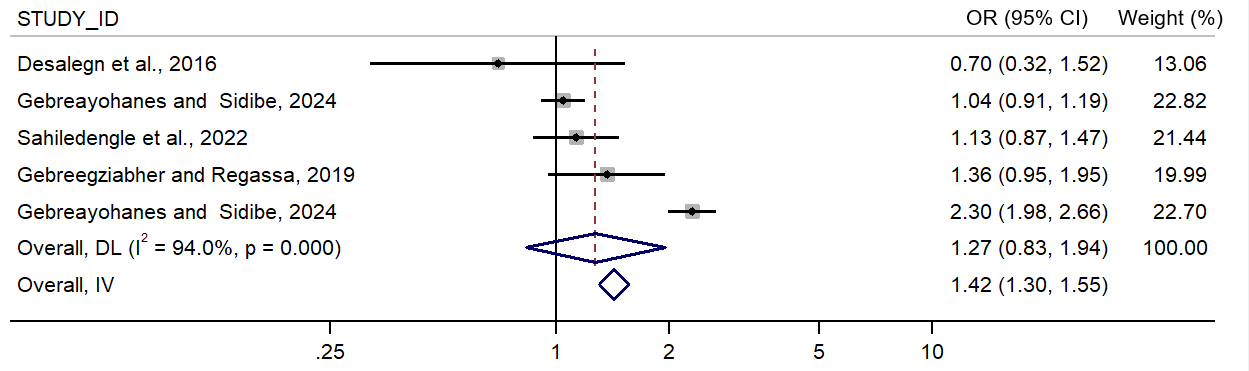

Supplement: Supplementary file 47 — forest suppl st. [file MCN-22-e70083-s007.tif]

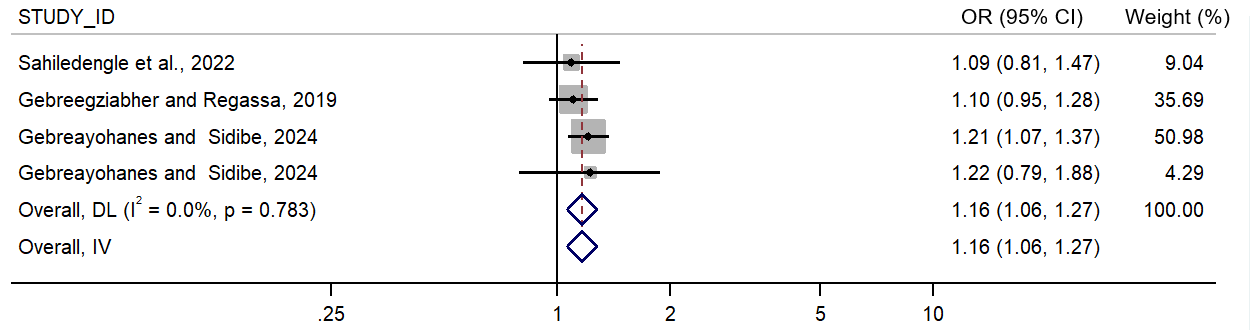

Supplement: Supplementary file 48 — forest suppl uw. [file MCN-22-e70083-s049.tif]

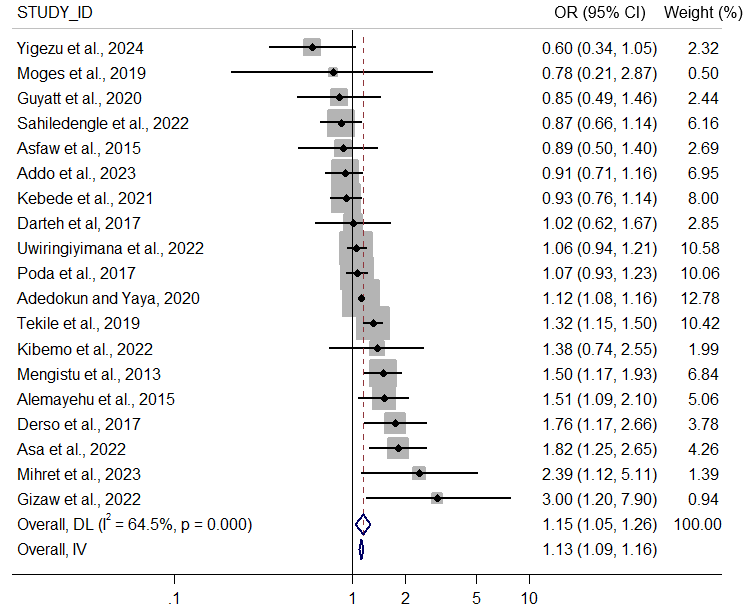

Supplement: Supplementary file 49 — forest toilet facility all. [file MCN-22-e70083-s010.tif]

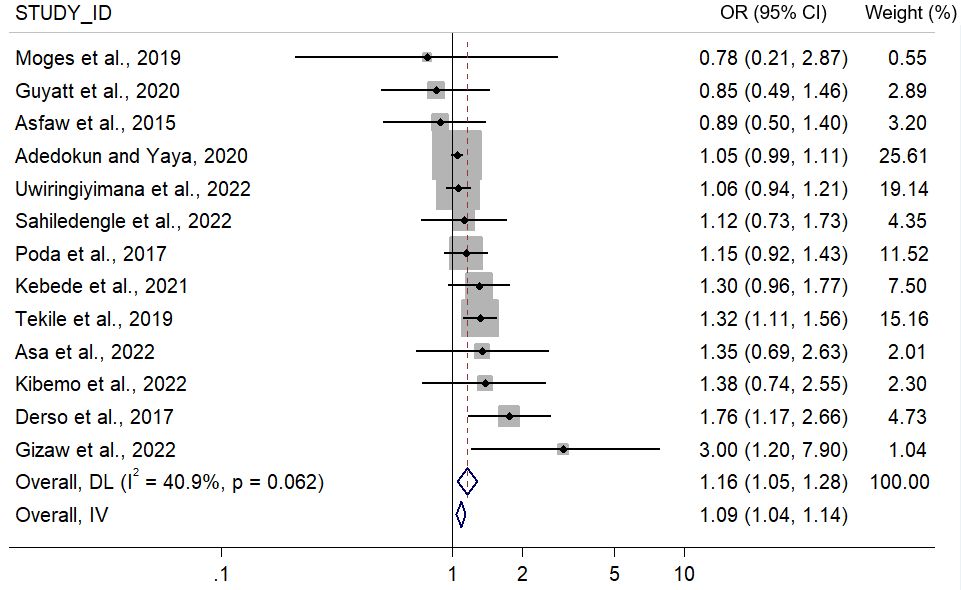

Supplement: Supplementary file 50 — forest toilet facility st. [file MCN-22-e70083-s032.tif]

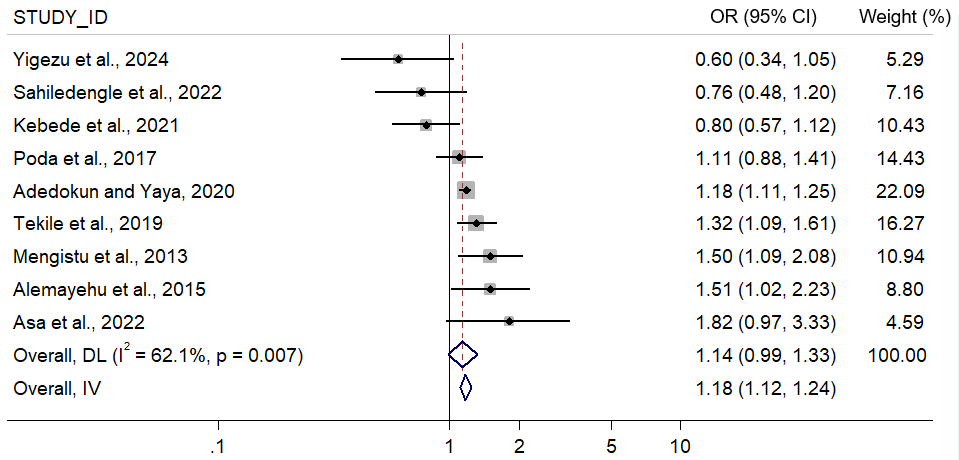

Supplement: Supplementary file 51 — forest toilet facility uw. [file MCN-22-e70083-s050.tif]

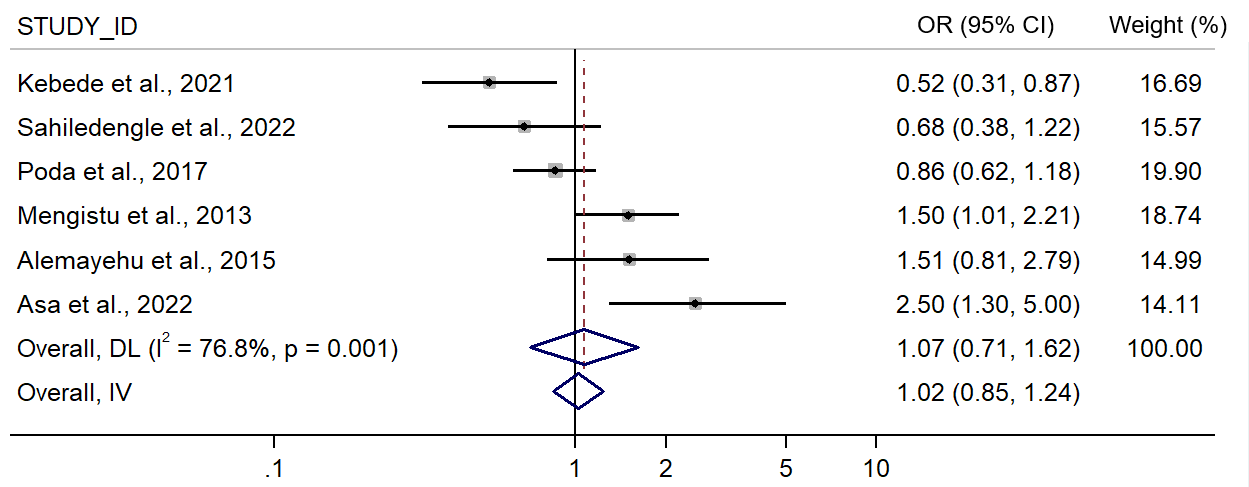

Supplement: Supplementary file 52 — forest toilet facility wt. [file MCN-22-e70083-s058.tif]

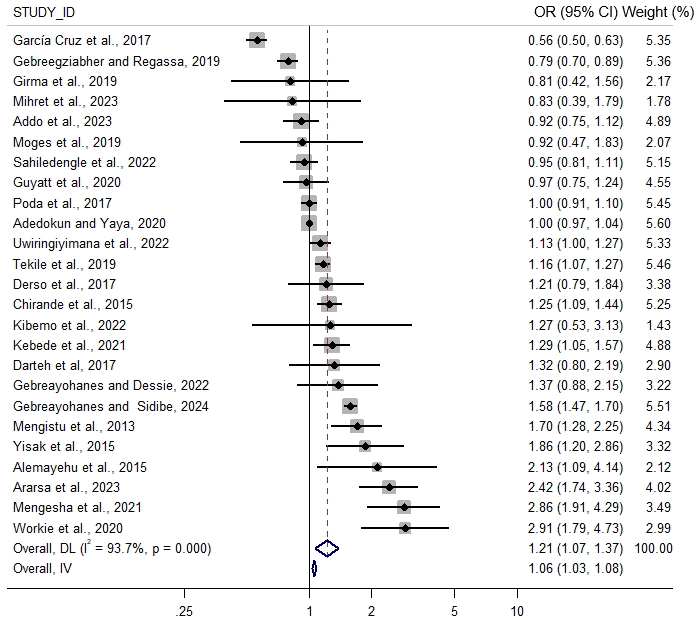

Supplement: Supplementary file 53 — forest water source all. [file MCN-22-e70083-s012.tif]

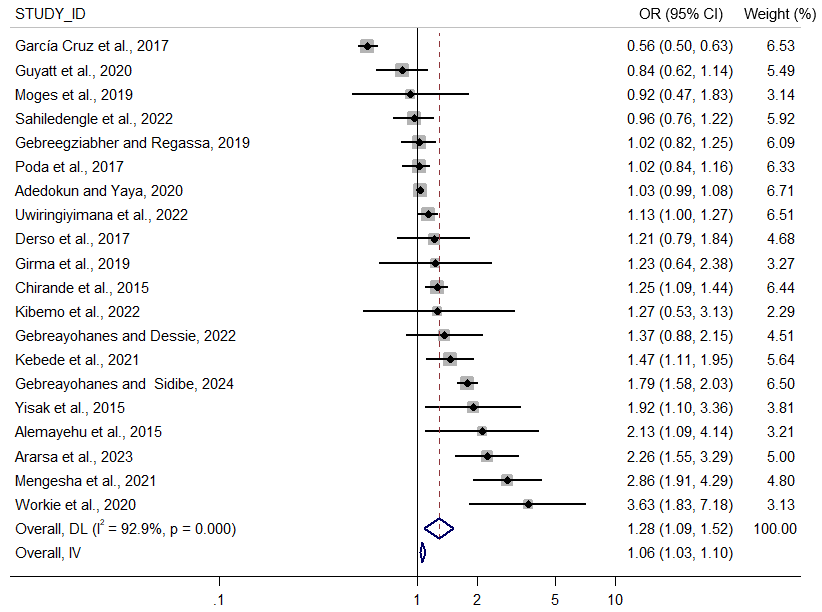

Supplement: Supplementary file 54 — forest water source st. [file MCN-22-e70083-s043.tif]

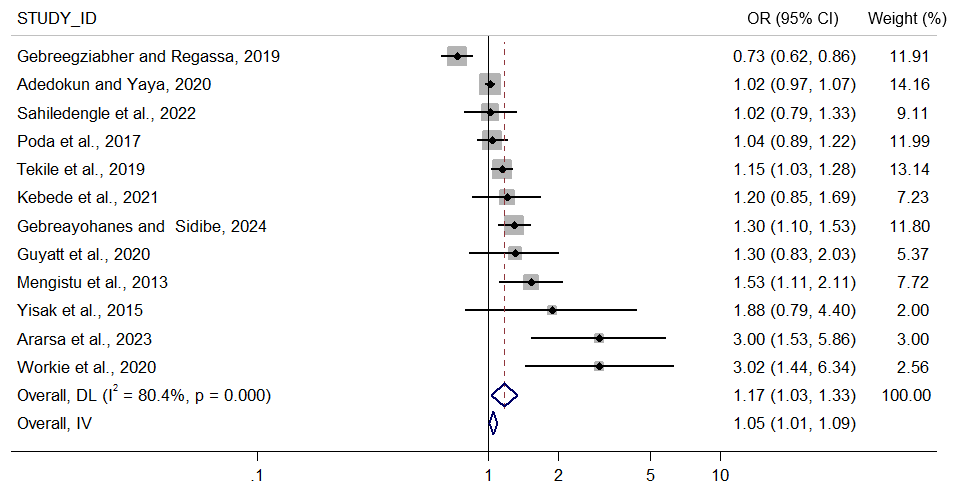

Supplement: Supplementary file 55 — forest water source uw. [file MCN-22-e70083-s016.tif]

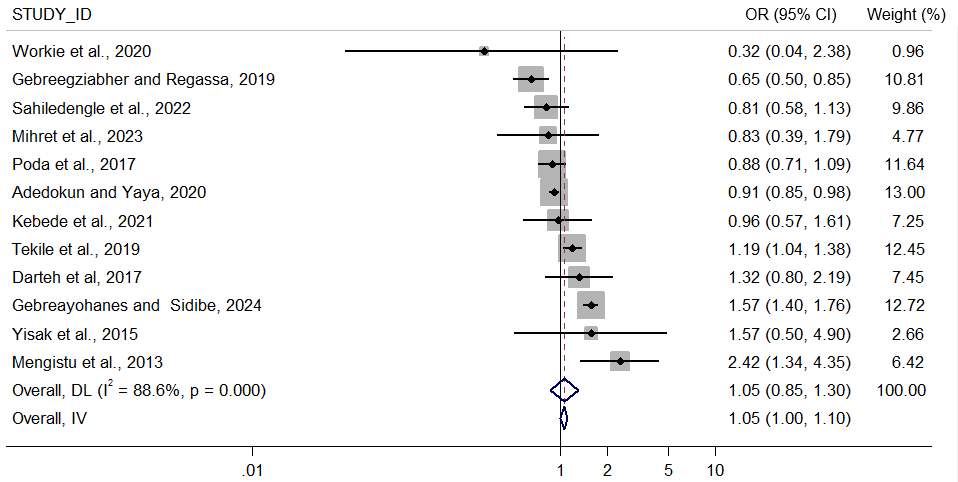

Supplement: Supplementary file 56 — forest water source wt. [file MCN-22-e70083-s015.tif]
