# Supplementary material for: Factors Associated With Childhood Undernutrition in Sub‐Saharan Africa: A Systematic Review and Meta‐Analysis
Source: Matern Child Nutr. 2025 Aug 31;22(1):e70083. doi: 10.1111/mcn.70083 (PMC12893520; doi:10.1111/mcn.70083)
Supplement: Supplementary file 57 — Suppl table 1 pattern of risk factors fin 10062025. [file MCN-22-e70083-s014.docx]

Supplementary Table 1. A matrix summarising the associations between risk factors and outcomes assessed

| Risk factors |  | Overall undernutrition |  | Stunting |  | Underweight |  | Wasting |  |
| --- | --- | --- | --- | --- | --- | --- | --- | --- | --- |
| *Child factors* |  |  |  |  |  |  |  |  |  |
| Anaemia |  | Yes |  | Yes |  | No |  | Yes |  |
| Low birthweight |  | Yes |  | Yes |  | Yes |  | Yes |  |
| No breastfeeding |  | No |  | No |  | No |  | No |  |
| Diarrhoea |  | Yes |  | Yes |  | Yes |  | Yes |  |
| Supplementation |  | No |  | No |  | Yes |  | No |  |
| *Household factors* |  |  |  |  |  |  |  |  |  |
| Rural residence |  | No |  | Yes |  | No |  | Yes |  |
| High family size |  | Yes |  | Yes |  | Yes |  | No |  |
| Food insecurity |  | No |  | No |  | Yes |  | No |  |
| Low maternal education |  | Yes |  | Yes |  | Yes |  | Yes |  |
| High number of children |  | Yes |  | No |  | Yes |  | No |  |
| Poor socioeconomic status |  | No |  | Yes |  | No |  | Yes |  |
| *Hygiene and sanitation* |  |  |  |  |  |  |  |  |  |
| Lack of handwashing |  | Yes |  | No |  | Yes |  | Yes |  |
| Unimproved toilet facility |  | No |  | No |  | No |  | No |  |
| Unimproved water source |  | No |  | No |  | Yes |  | No |  |
