# Supplementary material for: Factors Associated With Childhood Undernutrition in Sub‐Saharan Africa: A Systematic Review and Meta‐Analysis
Source: Matern Child Nutr. 2025 Aug 31;22(1):e70083. doi: 10.1111/mcn.70083 (PMC12893520; doi:10.1111/mcn.70083)
Supplement: Supplementary file 58 — Suppl table 2 quality scores. [file MCN-22-e70083-s001.docx]

Supplementary Table 1: Risk of bias of included studies

| Author | 1. Were the criteria for inclusion in the sample clearly defined? | 2. Were the study subjects and the setting described in detail? | 3. Was the exposure measured in a valid and reliable way? | 4. Were objective, standard criteria used for measurement of the condition? | 5. Were confounding factors identified? | 6. Were strategies to deal with confounding factors stated? | 7. Were the outcomes measured in a valid and reliable way? | 8. Was appropriate statistical analysis used? | Total score |
| --- | --- | --- | --- | --- | --- | --- | --- | --- | --- |
| Addo et al., 2023 | No | Yes | No | Unclear | Yes | No | Yes | Yes | 4 |
|  | No | No | Yes |  |  |  |  |  |  |
| Alemayehu et al., 2015 | No | Yes | Yes | Yes | Yes | No | Yes | Yes | 6 |
| Amare et al., 2016 | No | Yes | Yes | Yes | Yes | No | Yes | Yes | 6 |
| Ararsa et al., 2023 | Yes | Yes | Yes | Yes | Yes | Yes | Yes | Yes | 8 |
| Ariyo and Jiang, 2022 | Yes | Yes | Unclear | Unclear | Yes | No | Yes | Yes | 5 |
| Asa et al., 2022 | Yes | Yes | No | Yes | Yes | No | Yes | Yes | 6 |
| Asfaw et al., 2015 | Yes | Yes | Yes | Yes | Yes | Yes | Yes | Yes | 8 |
| Berhanu et al., 2018 | Yes | Yes | Unclear | Yes | Yes | Yes | Yes | Yes | 7 |
| Bliznashka et al., 2021 | Yes | No | Yes | Unclear | Yes | No | Yes | Yes | 5 |
| Chirande et al., 2015 | Yes | No | Unclear | Yes | Yes | Yes | Unclear | Yes | 5 |
| Derso et al., 2017 | Yes | Yes | Yes | Yes | Yes | No | Yes | Yes | 7 |
| Desalegn et al., 2016 | No | Yes | Yes | Yes | Yes | No | Yes | Unclear | 5 |
| Dewana et al., 2017 | No | Yes | Yes | Yes | Yes | Yes | Yes | Yes | 6 |
| Engidaye et al., 2022 | Yes | Yes | Unclear | Yes | Yes | No | Yes | Yes | 6 |
| Fekadu et al., 2015 | No | Unclear | Yes | Yes | Yes | No | Yes | Yes | 5 |
| García Cruz et al., 2017 | No | Yes | Yes | Yes | Yes | Yes | Yes | Yes | 7 |
| Gebreayohanes and Dessie, 2022 | Yes | Yes | Unclear | Yes | Yes | No | Yes | Yes | 6 |
| Gebreayohanes and Sidibe, 2024 | No | Yes | Unclear | Unclear | Yes | No | Yes | Yes | 4 |
| Gebreegziabher and Regassa, 2019 | No | Yes | Unclear | Unclear | Yes | Yes | Yes | Yes | 5 |
| Gebru et al., 2019 | No | No | Unclear | Yes | Yes | No | Yes | Yes | 4 |
| Girma et al., 2019 | Yes | Yes | Yes | Yes | Yes | No | Yes | Yes | 7 |
| Gizaw et al., 2022 | No | Yes | Yes | Yes | Yes | No | Yes | Yes | 6 |
| Guyatt et al., 2020 | No | Yes | Yes | Yes | Yes | No | Yes | Yes | 6 |
| Kebede et al., 2021 | Yes | Yes | Unclear | Unclear | Yes | No | Yes | Yes | 5 |
| Kibemo et al., 2022 | Yes | Yes | Yes | Yes | Yes | No | Yes | Yes | 7 |
| Mamabolo et al., 2005 | No | Yes | Yes | Yes | Yes | No | Yes | Yes | 6 |
| Matsungo et al., 2017 | Yes | Yes | Yes | Yes | Yes | Yes | Yes | Yes | 8 |
| Megabiaw and Rahman, 2013 | No | No | Unclear | Unclear | Yes | No | Yes | Yes | 3 |
| Menalu et al., 2021 | No | Yes | Yes | Yes | Yes | No | Yes | Yes | 6 |
| Mengesha et al., 2021 | No | Yes | Yes | Yes | Yes | No | Yes | Yes | 6 |
| Mengistu et al., 2013 | Yes | Yes | Yes | Yes | Yes | No | Yes | Yes | 7 |
| Mgongo et al., 2017 | No | Yes | Yes | Yes | Yes | No | Yes | Yes | 6 |
| Moges et al., 2019 | No | Yes | Yes | Yes | Yes | No | Yes | Yes | 6 |
| Ole Tankoi et al., 2016 | Yes | Yes | Yes | Yes | Yes | Yes | Yes | Yes | 8 |
| Poda et al., 2017 | No | Yes | Yes | Unclear | Yes | No | Unclear | Yes | 4 |
| Sahiledengle et al., 2022 | No | Yes | Unclear | Yes | Yes | Yes | Yes | Yes | 6 |
| Sserwanja et al., 2021 | No | Yes | Unclear | Yes | Yes | No | Yes | Yes | 5 |
| Teferi et al., 2016 | Yes | Yes | Yes | Yes | Yes | No | Yes | Yes | 7 |
| Tekile et al., 2019 | No | No | Unclear | Unclear | Yes | No | Yes | Yes | 3 |
| Uwiringiyimana et al., 2019 | No | Yes | Unclear | Yes | Yes | Yes | Yes | Yes | 6 |
| Uwiringiyimana et al., 2022 | No | Yes | Unclear | Unclear | No | No | Unclear | No | 1 |
| Workie et al., 2020 | Yes | Yes | Yes | Yes | Yes | No | Yes | Yes | 7 |
| Yisak et al., 2015 | No | Yes | Yes | Yes |  |  | Yes | Yes | 5 |
