# Supplementary material for: Factors Associated With Childhood Undernutrition in Sub‐Saharan Africa: A Systematic Review and Meta‐Analysis
Source: Matern Child Nutr. 2025 Aug 31;22(1):e70083. doi: 10.1111/mcn.70083 (PMC12893520; doi:10.1111/mcn.70083)
Supplement: Supplementary file 59 — Supplementary material 1 25042025. [file MCN-22-e70083-s039.docx]

**Main search string**

(((children) OR (infants) OR (children aged 6 - 24 months) OR (children aged 6 to 24 months) OR (children aged 6 - 59 months) OR (children aged 6 to 59 months) OR (children aged 0 - 59 months) OR (children aged 0 to 59 months) OR (children aged 0 - 60 months) OR (children aged 0 to 60 months) OR (under five years children) OR (children under five years) OR (under five children) OR (under 5 years children) OR (children under 5 years) OR (under 5 children)) OR (U5C) OR (CU5) OR (Children below five years of age)) AND ((low and middle-income countries) OR (low and middle-income countries) OR (LMIC) OR (developing countries) OR (Sub-Saharan African countries) OR (Sub-Saharan Africa) OR (SSA) OR (Africa) NOT (ASIA)) AND ((Underweight) OR (stunting) OR (Wasting) OR (undernutrition) OR (malnutrition)) AND ((cross-sectional) OR (cross sectional) OR (cross-sectional studies) OR (cross sectional studies) OR (cohort) OR (cohort studies) OR (case-control) OR (case-control studies) OR (case control) OR (case control studies) OR (survey) OR (demographic and health survey) AND (health and demographic surveillance system) AND (national survey) OR (demographic and health survey) OR (health and demographic surveillance system) OR (national survey) AND (demographic and health survey data) AND (health and demographic surveillance system data) AND (national survey data) OR (demographic and health survey data) OR (health and demographic surveillance system data) OR (national survey data) NOT (randomized controlled trials) NOT (RCT) NOT (intervention) NOT (experimental) NOT (retrospective))

) AND (("2000/01/01"[Date - Publication] : "3000"[Date - Publication]))

**Search string with SSA countries regions and individual countries**

(((children) OR (infants) OR (children aged 6 - 24 months) OR (children aged 6 to 24 months) OR (children aged 6 - 59 months) OR (children aged 6 to 59 months) OR (children aged 0 - 59 months) OR (children aged 0 to 59 months) OR (children aged 0 - 60 months) OR (children aged 0 to 60 months) OR (under five years children) OR (children under five years) OR (under five children) OR (under 5 years children) OR (children under 5 years) OR (under 5 children)) OR (U5C) OR (CU5) OR (Children below five years of age)) AND ((low and middle-income countries) OR (low and middle-income countries) (LMICs) OR (developing countries) OR (Sub-Saharan African countries) OR (Sub-Saharan Africa) OR (SSA) OR (Africa) OR (Central Africa) OR (East Africa) OR (West Africa) OR (Southern Africa) OR(Angola) OR (Benin) OR (Botswana) OR (Burkina Faso) OR (Burundi) OR (Cabo Verde) OR (Cameroon) OR (Central African Republic) OR (Chad) OR (Comoros) O (Congo) OR (Côte d'Ivoire) OR (Democratic Republic of the Congo) OR (Djibouti) OR (Equatorial Guinea) OR (Eritrea) OR (Eswatini) OR (Ethiopia) OR (Gabon) OR (Gambia) OR (Ghana) OR (Guinea) OR (Guinea-Bissau) OR (Kenya) OR (Lesotho) OR (Liberia) OR (Madagascar) OR (Malawi) OR (Mali) OR (Mauritania) OR (Mauritius) OR (Mozambique) OR (Namibia) OR (Niger) OR (Nigeria) OR (Rwanda) OR (Sao Tome and Principe) (Senegal) OR (Seychelles) OR (Sierra Leone) OR (Somalia) OR (South Africa) OR (South Sudan) OR (Sudan) OR (Togo) OR (Tanzania) (Uganda) OR (Zambia) OR (Zimbabwe) NOT ((ASIA)) AND ((Underweight) OR (stunting) OR (Wasting) OR (undernutrition) OR (malnutrition)) AND ((cross-sectional) OR (cross sectional) OR (survey) OR (demographic and health survey) AND (health and demographic surveillance system) AND (national survey) OR (demographic and health survey) OR (health and demographic surveillance system) OR (national survey) AND (demographic and health survey data) AND (health and demographic surveillance system data) AND (national survey data) OR (demographic and health survey data) OR (health and demographic surveillance system data) OR (national survey data) NOT (randomized controlled trials) NOT (RCT) NOT (intervention) NOT (experimental) NOT (retrospective))) AND

(("2000/01/01"[Date - Publication] : "3000"[Date - Publication]))
